# Eye tracking identifies biomarkers in α-synucleinopathies versus progressive supranuclear palsy

**DOI:** 10.1007/s00415-022-11136-5

**Published:** 2022-04-30

**Authors:** Mahboubeh Habibi, Wolfgang H. Oertel, Brian J. White, Donald C. Brien, Brian C. Coe, Heidi C. Riek, Julia Perkins, Rachel Yep, Laurent Itti, Lars Timmermann, Christoph Best, Elisabeth Sittig, Annette Janzen, Douglas P. Munoz

**Affiliations:** 1 Department of Neurology, Philipps-University Marburg, 35043 Marburg, Germany; 2 Centre for Neuroscience Studies, Queen’s University, 18 Stuart Street, Kingston, ON K7L 3N6 Canada; 3 Department of Biomedical and Molecular Sciences, Queen’s University, Kingston, ON Canada; 4 Department of Computer Science, University of Southern California, Los Angeles, CA USA

**Keywords:** Parkinson’s disease, REM sleep behaviour disorder, Multiple system atrophy, Progressive supranuclear palsy, Eye movement, Alpha-synucleinopathy, Biomarker

## Abstract

**Objectives:**

This study (1) describes and compares saccade and pupil abnormalities in patients with manifest alpha-synucleinopathies (αSYN: Parkinson’s disease (PD), Multiple System Atrophy (MSA)) and a tauopathy (progressive supranuclear palsy (PSP)); (2) determines whether patients with rapid-eye-movement sleep behaviour disorder (RBD), a prodromal stage of αSYN, already have abnormal responses that may indicate a risk for developing PD or MSA.

**Methods:**

Ninety (46 RBD, 27 PD, 17 MSA) patients with an αSYN, 10 PSP patients, and 132 healthy age-matched controls (CTRL) were examined with a 10-min video-based eye-tracking task (Free Viewing). Participants were free to look anywhere on the screen while saccade and pupil behaviours were measured.

**Results:**

PD, MSA, and PSP spent more time fixating the centre of the screen than CTRL. All patient groups made fewer macro-saccades (> 2^◦^ amplitude) with smaller amplitude than CTRL. Saccade frequency was greater in RBD than in other patients. Following clip change, saccades were temporarily suppressed, then rebounded at a slower pace than CTRL in all patient groups. RBD had distinct, although discrete saccade abnormalities that were more marked in PD, MSA, and even more in PSP. The vertical saccade rate was reduced in all patients and decreased most in PSP. Clip changes produced large increases or decreases in screen luminance requiring pupil constriction or dilation, respectively. PSP elicited smaller pupil constriction/dilation responses than CTRL, while MSA elicited the opposite.

**Conclusion:**

RBD patients already have discrete but less pronounced saccade abnormalities than PD and MSA patients. Vertical gaze palsy and altered pupil control differentiate PSP from αSYN.

**Supplementary Information:**

The online version contains supplementary material available at 10.1007/s00415-022-11136-5.

## Introduction

Assessment of the oculomotor system is an essential part of the neurological examination, especially for the differential diagnosis of neurodegenerative movement disorders such as alpha-synucleinopathies (αSYN) – Parkinson’s disease (PD) [1], dementia with Lewy bodies (DLB), multiple system atrophy (MSA) [2] and the tauopathy (TAU) progressive supranuclear palsy (PSP). It is often difficult to clearly differentiate PSP from αSYN early in the disease process, particularly when atypical characteristics are present [3–5]. Video-based eye tracking can reliably and objectively measure different saccade, and pupil behaviour to assess the intactness of cortical and subcortical neural circuits and, therefore, potentially confirm clinical diagnosis and improve the oculomotor assessment and accuracy. With the advent of potentially neuroprotective therapies to treat αSYN and TAU, changes of saccade and pupil behaviour components in prodromal disease stages are of significant interest and may eventually qualify as prodromal biomarkers or even progression markers.

In this respect, isolated rapid eye movement (REM) sleep behaviour disorder (RBD) is a distinct prodromal stage of the manifest αSYN: within 15 years, up to 85% of RBD patients will convert to either PD, DLB, or more rarely MSA [6]. Therefore, RBD is suitable for looking for PD, DLB, and MSA prodromal markers. In the manifest stage, early autonomic dysfunctions are the key clinical parameters in MSA that differentiate MSA from PD [2, 8]. Various studies have compared the saccadic alterations in PD and MSA [9–12]. However, a comprehensive comparative study assessing changes in saccade and pupil behaviour in the two manifest αSYNs (PD and MSA) versus the prodromal αSYN RBD has not been done.

In this study, we not only compare various αSYNs, but also contrast them to the tauopathy PSP, an atypical parkinsonian disorder that is, for example, pathologically differentiable from PD by symmetrical tissue loss in the frontal cortex [13]. PSP is, in particular, characterized by impaired oculomotor control [14–16], which is a key symptom in many PSP patients [17]. Individuals with PSP show reduced vertical saccade frequency, saccade amplitude, and saccade velocity compared to age-matched controls (CTRL) [17–20]. Because of the difficulties with the early differential diagnosis between PD, MSA, and PSP, we devised a simple video-based eye tracking task, called Free Viewing (FV), to determine whether there are reliable differences in saccade and/or pupil control in PSP versus the αSYNs [7].

Previous studies have used structured tasks to identify abnormal saccade responses in neurodegenerative diseases [21–24]. Here, we employ the simple FV paradigm in which patients are shown a series of short video clips on a computer screen, and they are free to view these clips however they choose. This approach does not allow for a detailed assessment of saccade dysmetria, but it allows for a richer assessment of saccade and pupil behaviour to be recorded in a dynamic visual setting with a high temporal and spatial resolution to reveal abnormalities. Most importantly, this setting does not require extensive preparatory instructions for the participant to perform the task. We use the FV paradigm for the investigation of oculo- and pupillomotor function in the prodromal (RBD) and manifest stages of αSYN (in this study PD and MSA) in comparison to PSP which is a tauopathy with well-known oculomotor deficits. We specifically address the following questions: (1) which saccade or pupil parameters – when captured with FV—are altered in patients with the manifest αSYN PD and MSA or the tauopathy PSP? (2) Using these parameters, does the FV paradigm allow to differentiate between patients with αSYN and PSP? (3) Are abnormal pupil and saccade responses observed in PD or MSA also detectable in the prodromal αSYN stage RBD?

## Materials and methods

### Participants

We included five different groups of participants. Patients diagnosed with PD, MSA, RBD, and PSP were recruited in the Department of Neurology, Philipps-University Marburg, Germany. CTRL subjects were recruited as part of a large study within the Faculty of Health Sciences at Queen’s University in Kingston, Canada. The study protocol was approved by the human research ethics board of the Faculty of Medicine, Philipps-University Marburg (Protocol ID: 147/16) and the Faculty of Health Sciences, Queen’s University (Protocol ID: PHYS-007-97; CNS-005-10). Voluntary informed consent was obtained from each participant after a verbal and written explanation of the study, according to the Declaration of Helsinki.

All patients recruited were 45–84 years of age (see for Exclusion criteria Supplementary Material). All patients underwent clinical testing with the Montreal Cognitive Assessment (MoCA) [25], Unified PD Rating Scale (UPDRS-III and/or Movement Disorder Society (MDS)-UPDRS scale III), Beck’s Depression Inventory-II (BDI-II) [26], PD Non-Motor Scale (PDNMS) [27], and the REM Sleep Behaviour Disorder Screening Questionnaire (RBDSQ) [28]. Clinical and demographic data are provided in Supplementary Material (Supplementary Table 1).

*RBD*. Forty-six patients (5 females, 41 males, age range: 50.6–76.4 years) with video-polysomnography-confirmed RBD (Darien IL, AASM, 2014) had mean UPDRS-III, MoCA, and BDI-II scores equal to 1.61, 28.2, and 7.7, respectively. All RBD patients were interviewed for a medical and drug history in detail and received a complete neurological examination. This procedure was repeated twice over a period of 1 year to reduce the risk of including subjects with secondary RBD in the study. In addition, we excluded RBD patients with cognitive impairment (MoCA < 25), and this would presumably minimize the number of patients likely to convert to DLB [29].

*PD*. PD patients were diagnosed according to the United Kingdom Brain Bank Criteria. Twenty-seven PD patients (2 females, 25 males, age range: 45.7–84.1 years) were included: 7 PD patients were de novo PD patients, 3 PD patients were investigated under treatment with dopaminergic medication (on-state), 14 PD patients were at least 12 h without medication (defined off-state), and three with unknown medication status. Given the relatively minor variation in saccadic behaviour between on and off states, all three groups were pooled into a single PD group, as previously reported [30]. Mean UPDRS-III, MoCA, and BDI-II scores for PD were 15.7, 27.8, and 8.4, respectively.

*MSA*. Seventeen MSA patients (seven females, ten males, age range: 51.6—73.8 years) were diagnosed according to the second consensus statement on the diagnosis of MSA [8]. Mean UPDRS-III, MoCA, and BDI-II scores in MSA were 27.4, 26.7, and 11.0, respectively.

*PSP*. Ten PSP patients (five females, five males, age range: 62.5–82.2 years) were diagnosed according to the National Institute of Neurological Disorders and Stroke and the Society for PSP (NINDS-SPSP) and Höglinger et al. [15] criteria. PSP patients showed severe motor and cognitive problems with mean UPDRS-III, MoCA, and BDI-II scores of 34.7 and 20.8, and 16.5, respectively.

*Control participants (CTRL)*. One hundred thirty-two healthy age-matched CTRL participated in the study (86 female, 46 male, age range: 45.5–84.3 years). Age is known to influence many saccade parameters (e.g., increased saccade latency, decreased saccade frequency, decreased saccade amplitude, and velocity) [31–33]. To control for age effects, we created a separate CTRL group for each patient group. For each group, we selected CTRL that had a maximum of ± 1 year age difference with each patient (Supplementary Fig. 1). We confirmed that each control group was matched in age to its corresponding patient group. The CTRL groups, therefore, had different numbers and overlapping individuals in each group.

### Eye tracking task

Participants were seated with their head resting on a chinrest and a forehead rest so that their eyes were positioned 60 cm away from a computer screen in a dark, windowless room, with a curtain drawn between them and the operator to limit any potential distractions. Despite this, PSP patients occasionally made a backward head movement during the eye tracking. To prevent this from happening again, an experimenter used their hands to keep their head in a stable position on the chin and forehead rest. Additionally, the participants were seated in a chair which included a backrest to keep them from falling backward. Occasionally, we used a pillow to bridge the space between their neck and the backrest of the chair. We attempted to keep the amount of head motion to a minimum while collecting the data. Additionally, if participants pushed back, the eye tracker stopped recording, and the task was recalibrated. All data were collected using a video-based eye tracker (Eyelink 1000 Plus; SR Research, Mississauga, Ontario, Canada), recording monocular right pupil size and eye position at 500 Hz (Details in Supplementary).

### Visual stimuli

Videos were displayed on a 17-inch LCD monitor, and all participants viewed a total of ten movies (Supplementary Fig. 2A). Each movie was approximately 1 min in duration and consisted of 15–17 video clips that were ~ 2–5 s in duration (mean = 3.76, mode = 4). We made the video clips of scenes with and without humans, animals, buildings, cars, and the clips were randomly assembled so that viewing was similar to watching television and changing the channel every few seconds. The clips were presented in a fixed sequence within each movie, but the order of the ten movies was randomized between participants. The task required no instruction; the participants simply viewed the video clips. Clip changes produced a large visual perturbation that stimulated much of the central retina, producing a large visual transient signal [34] carried to all central visual areas that altered ongoing saccade and pupil behaviour.

### Saccade analysis

We divided the analyses into: (1) low-level statistics independent of video content, and (2) analyses aligned on clip changes (see Supplementary Fig. 2B). Auto-marking scripts developed in MATLAB were used to classify each trial and all eye movements (saccades, fixations, and pupil size). All saccades were marked for direction, amplitude, peak velocity, and duration [35]. We defined macro-saccades as all saccades ≥ 2° amplitude and micro-saccades [36–41] as all saccades < 2° amplitude.

The coordinates of each fixation were used to create gaze distribution maps (see Supplementary Materials for details). Centre bias, the excessive time gazing at the centre of the screen [7], was calculated for each participant and was defined as the mean ± 5º around the centre of the gaze distribution map for each participant.

We computed the frequency (saccade-count/viewing-duration) and average saccade amplitude in each of 60 different saccade directions (each bin was 6° polar angle). In subsequent analysis, we separated horizontal and vertical saccades because PSP patients have vertical gaze impairments specifically [42]. All saccades with direction ± 45° of the horizontal meridian were defined as horizontal, and all saccades ± 45° of the vertical meridian were defined as vertical.

There is a fundamental relationship between the amplitude and peak velocity of saccades known as the main sequence [43], which measures the integrity of the brainstem saccade premotor circuit [44]. We measured the amplitude and peak velocity of all saccades > 2° and plotted peak velocity as a function of log amplitude for each participant, which produces a linear relationship [43]. We then fit a linear function to the resulting data (Supplementary Fig. 3).

The clip transitions produced transient changes in saccade and pupil behaviour. We computed the macro- and micro-saccade rate (saccades/s) for each participant using a peri-stimulus time histogram (PSTH, 2 ms bin width due to the 500 Hz sample rate; see Supplementary Materials). For macro-saccades, we computed a baseline rate for each participant ( – 200 to + 50 ms relative to the clip change), as well as the magnitude and timing of the dip in macro-saccade rate (“saccade suppression” [45]), the peak macro-saccade rate after clip transition (maximum value from the time of suppression to 300 ms post clip change), and the steady state macro-saccade rate (averaged from 1000 to 3000 ms after clip change).

A similar set of micro-saccade parameters was extracted for each participant. Micro-saccade PSTHs were created, and we computed a baseline rate (average rate from  – 200 to + 50 ms relative to clip change). We computed the magnitude and timing of the suppression in micro-saccade rate in the epoch from 70 to 400 ms after clip change. We computed the steady state micro-saccade rate, which was the average over an epoch 1000–3000 ms after clip change.

### Pupil analysis

We measured the mean global luminance of every frame of every movie by computing the luminance gamma functions of the red, green, and blue color gamuts at various output levels. We then used those functions to compute the luminance of every pixel in the frame and averaged across all pixels to get the mean screen luminance for that frame. We correlated the mean pupil size with the mean screen luminance (cd/m^2^) across clips for each participant (Supplementary Fig. 5A). For each participant, we extracted the y-intercept and slope (Supplementary Fig. 5B and 5C).

The clip changes produced luminance changes that impacted pupil size. We measured this luminance change and ranked all clip transitions to extract the 30 clip changes with the greatest increase in luminance and the 30 clip changes with the greatest decrease in luminance to measure the impact of clip change on pupil behaviour (see Supplementary).

Finally, we tested the correlation of all eye movement parameters versus UPDRS-III scores to examine the relationship between the severity of the motor dysfunction and oculomotor and pupillometry parameters.

### Statistical analysis

All statistical comparisons were performed in MATLAB using a pairwise non-parametric test, Mann–Whitney-*U*-test, to determine the significant statistics. Multiple comparisons adjustments were excluded due to the exploratory aspect of the study. We performed different statistical comparisons to address our main questions. First, we compared patients to CTRL. We consistently report the patient values followed by CTRL unless stated otherwise. We then compared across patient groups to first determine if the prodromal αSYN group RBD started to already present abnormalities which were identified in PD and MSA, and then to identify which abnormalities reliably differentiated PSP from the αSYN groups.

## Results

### Low-level saccade statistics

#### Gaze distribution maps

**Fig. 1:**
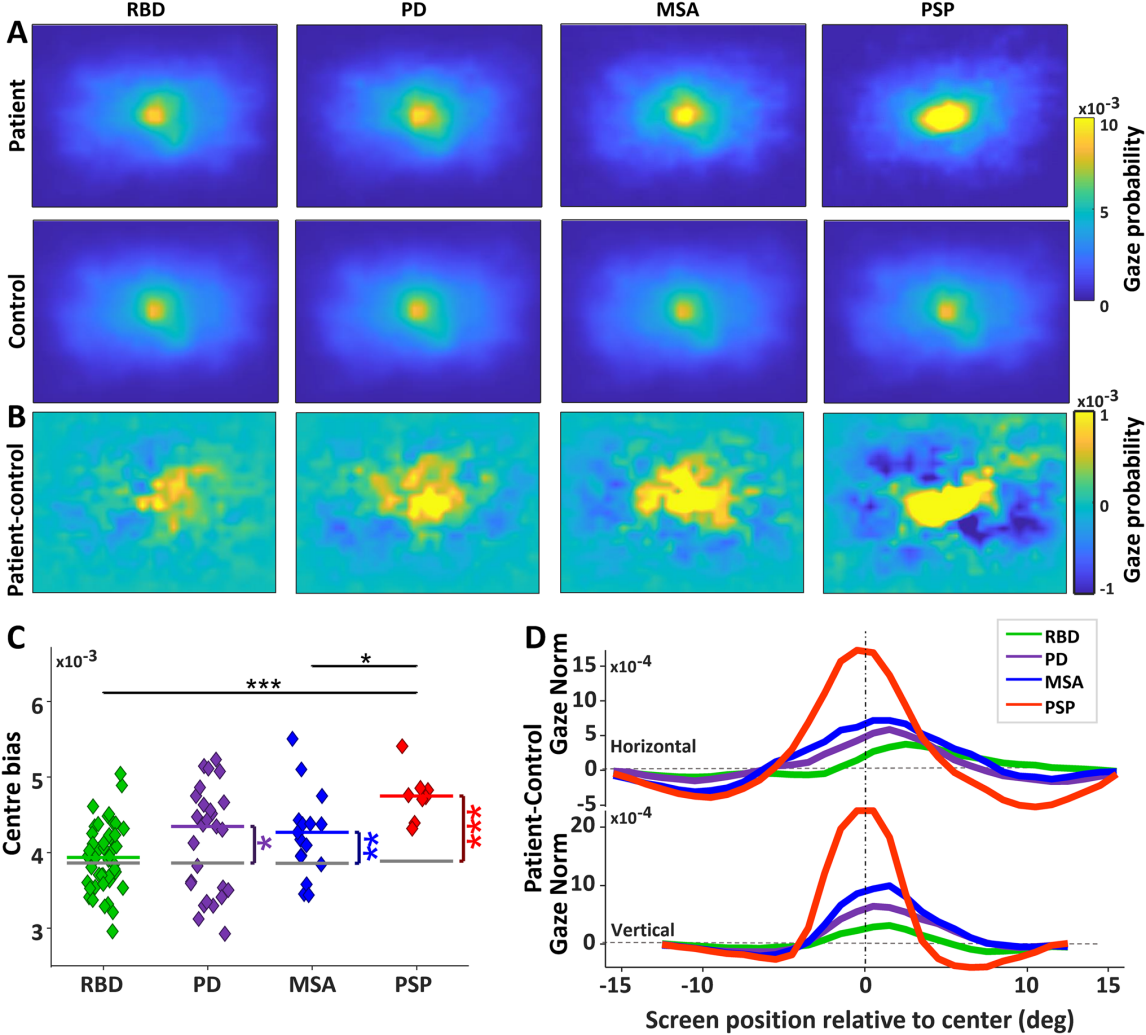
Characteristics of gaze distribution. **A** Gaze distribution for each group. The screen spanned 32 deg horizontally and 26 deg vertically. Higher gaze probability is represented by yellow. **B** Difference gaze probability maps of the patients minus controls, with yellow (positive values) indicating higher gaze probability for patients than controls. **C** Individual values of centre bias, which was defined as the value at the centre of the gaze probability map in A for each participant. The gray horizontal lines indicate the CTRL group’s median, and the colorful horizontal lines indicate the patient groups’ median. Comparisons between the patients and CTRL were shown with vertical lines with asterisks if significant. Horizontal bares with asterisks indicate comparison between the disease groups. **D** Difference in gaze probability between each patient group and their respective control group, extracted from a slice through the horizontal and vertical meridian of the difference gaze probability maps in C (positive values indicate higher gaze probability for patients relative to controls). Asterisks show a significance level of *P < .05 and **P < .01 and *** *P* < .001(same in all further figures). *RBD* REM sleep behaviour disorder, *PD* Parkinson’s disease, *MSA* Multiple system atrophy, *PSP* Progressive supranuclear palsy

We first analysed the distributions of all fixations for the 10 min of FV from all participants, which produced gaze distribution maps. Patient groups (Fig. 1A, top row) and their corresponding CTRL groups (Fig. 1A, bottom row) had a strong centre bias (indicated in yellow), spending most of their time fixating on locations around screen’s centre [7]. We subtracted the gaze distribution maps of CTRL groups from the patient groups to reveal the differences in the centre bias (Fig. 1B). PD, MSA and PSP groups had a significantly greater centre bias than CTRL (Fig. 1C; PD: 0.0043 average gaze/visual degree versus 0.0039, *P* < 0.05, MSA: 0.0042 versus 0.0039, *P* < 0.01, PSP: 0.0047 versus 0.0039, *P* < 0.0001). That means patient groups spent less time exploring the peripheral parts of the video clips than CTRL. We then compared the patient groups to one another. RBD and MSA had a significantly smaller centre bias than PSP (RBD versus PSP: *P* < 0.001, MSA versus PSP: *P* < 0.05). We also looked at the difference in gaze distributions between patients and controls along the horizontal and vertical meridians (Fig. 1D; patient-CTRL). The PSP group had a greater centre bias along horizontal and vertical meridians compared to all other groups.

#### Saccade and fixation duration distributions

**Fig. 2:**
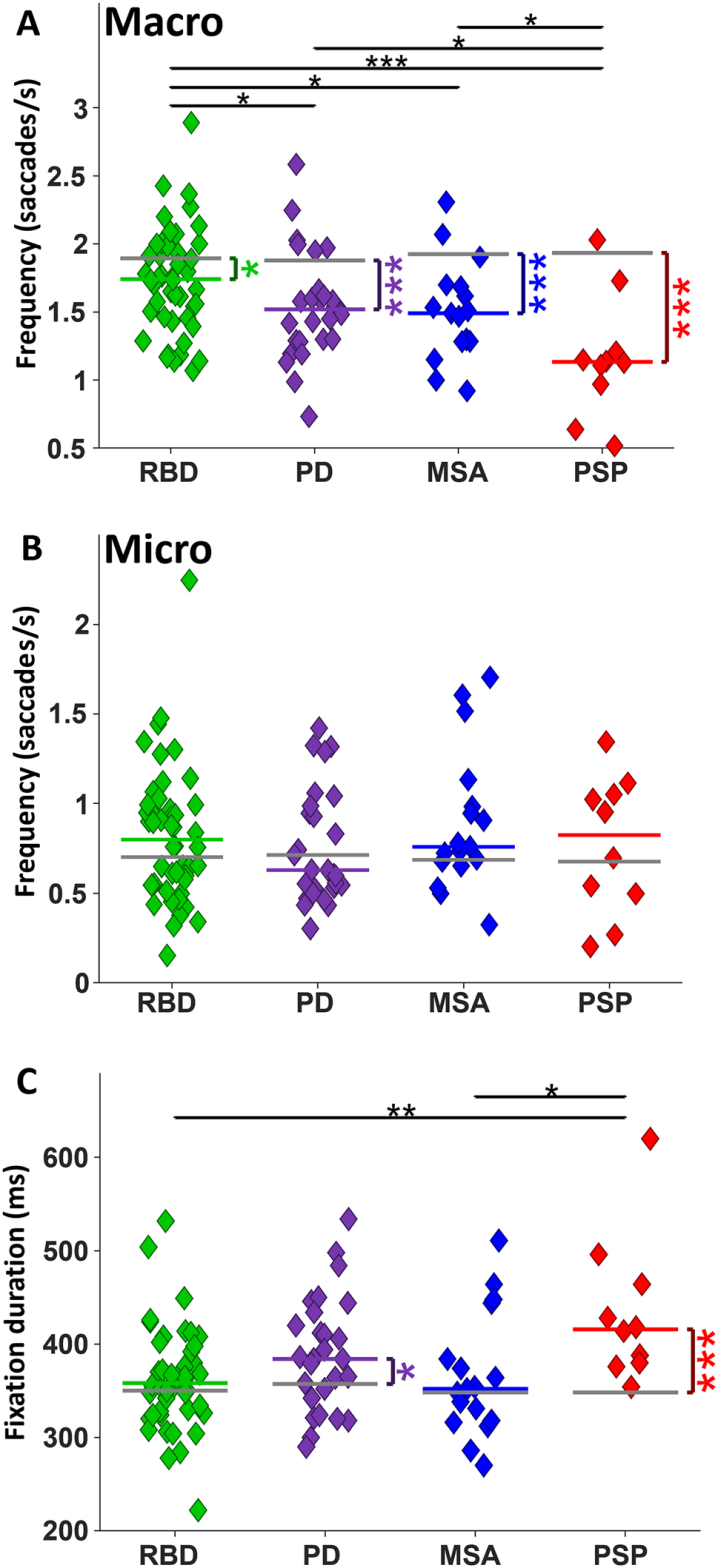
Saccade frequency and median fixation duration. **A** Macro-saccade rate per second for each group. The most important finding is the difference between RBD and PD. **B** Micro-saccade rate per second. **C** Median Fixation duration of each group

We computed low-level statistics of saccade frequency, direction, and amplitude, as well as fixation durations. For these analyses, we separated macro-saccades from micro-saccades. All patient groups made fewer macro-saccades than CTRL (Fig. 2A; RBD: 1.74 saccades/s versus 1.89, *P* < 0.05; PD: 1.51 versus 1.87, *P* < 0.0001; MSA: 1.49 versus 1.92, *P* < 0.0001; PSP: 1.13 versus 1.94, *P* < 0.0001). Among patient groups, RBD had a higher macro-saccade frequency, not only relative to PD (*P* < 0.05) and MSA (*P* < 0.05) but also relative to PSP (*P* < 0.001). Both PD and MSA had a higher macro-saccades rate relative to PSP (both *P* < 0.05). The overall micro-saccade rate (Fig. 2B) was not significantly different across groups. As a direct result of fewer macro-saccades, PSP and PD had longer fixation durations than CTRL (Fig. 2C; PD: 384 ms versus 357, *P* < 0.05; PSP: 416 versus 348, *P* < 0.001). PSP also had significantly longer fixation durations than RBD (*P* < 0.01) and MSA (*P* < 0.05).

#### Distribution of macro- and micro-saccade directions

**Fig. 3:**
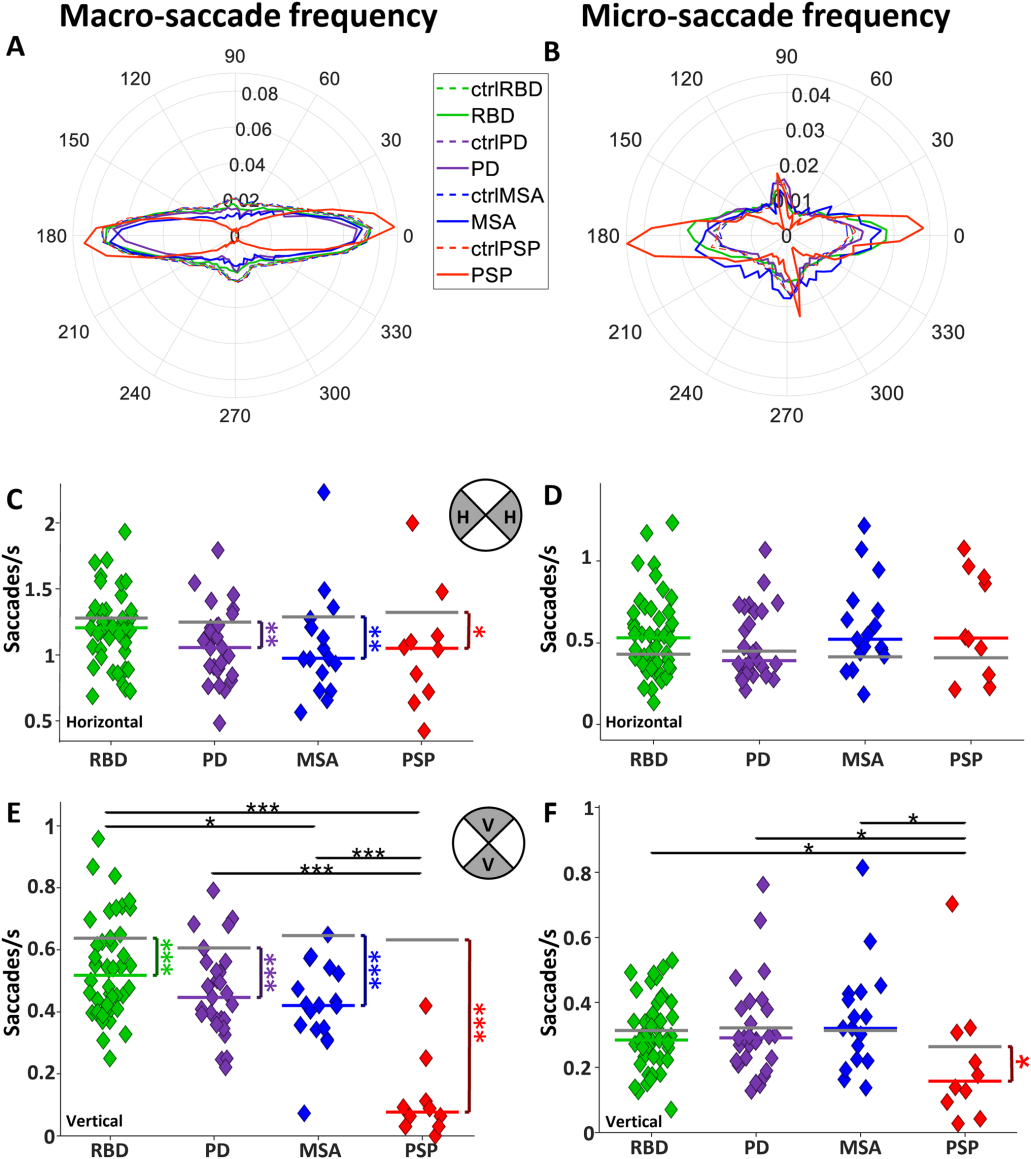
Saccade rate in different directions. **A** Polar histogram of macro-saccades frequency and **B** polar histogram of micro-saccades frequency for every group. Polar coordinates are saccade directions, and each circle represents the average macro/micro-saccade frequency within each group. **C** and **D** Horizontal macro and micro-saccade frequency, respectively. **E** and **F** vertical macro and micro-saccade frequency of each individual, respectively

PSP patients develop vertical gaze palsy during disease progression [46]. To determine if there were directional biases in the distribution of saccade directions, we computed the frequency of macro-and micro-saccades in 60 different directions (Fig. 3 A–B). PD, MSA, and PSP had reduced horizontal macro-saccade frequency compared to CTRL, but RBD did not differ from CTRL (Fig. 3C; RBD: 1.21 saccades/s versus 1.28, *P* = 0.08; PD: 1.05 versus 1.25, *P* < 0.01; MSA: 0.97 versus 1.28, *P* < 0.01; PSP: 1.05 versus 1.31, *P* < 0.05). Overall micro-saccade frequency in the horizontal direction did not differ between patient groups and CTRL (Fig. 3D). Vertical macro-saccades were reduced in all patient groups relative to CTRL (Fig. 3E, RBD: 0.51 saccades/s versus 0.63, *P* < 0.001, PD: 0.44 versus 0.60, *P* < 0.0001, MSA: 0.42 versus 0.64, *P* < 0.0001, and PSP: 0.07 versus 0.62, *P* < 0.0001). Comparisons among αSYN groups revealed a significant difference between RBD and MSA (*P* < 0.05), while all αSYN groups had more vertical macro-saccades than PSP (all *P* < 0.001). PSP displayed lower vertical micro-saccade frequency than CTRL (Fig. 3F, RBD: 0.28 saccades/s versus 0.31, *P* = 0.39; PD: 0.29 versus 0.32, *P* = 0.28; MSA: 0.32 versus 0.31, *P* = 0.77; PSP:0.15 versus 0.26, *P* < 0.05) and lower than all patient groups (all *P* < 0.05).

#### Saccade amplitude

**Fig. 4:**
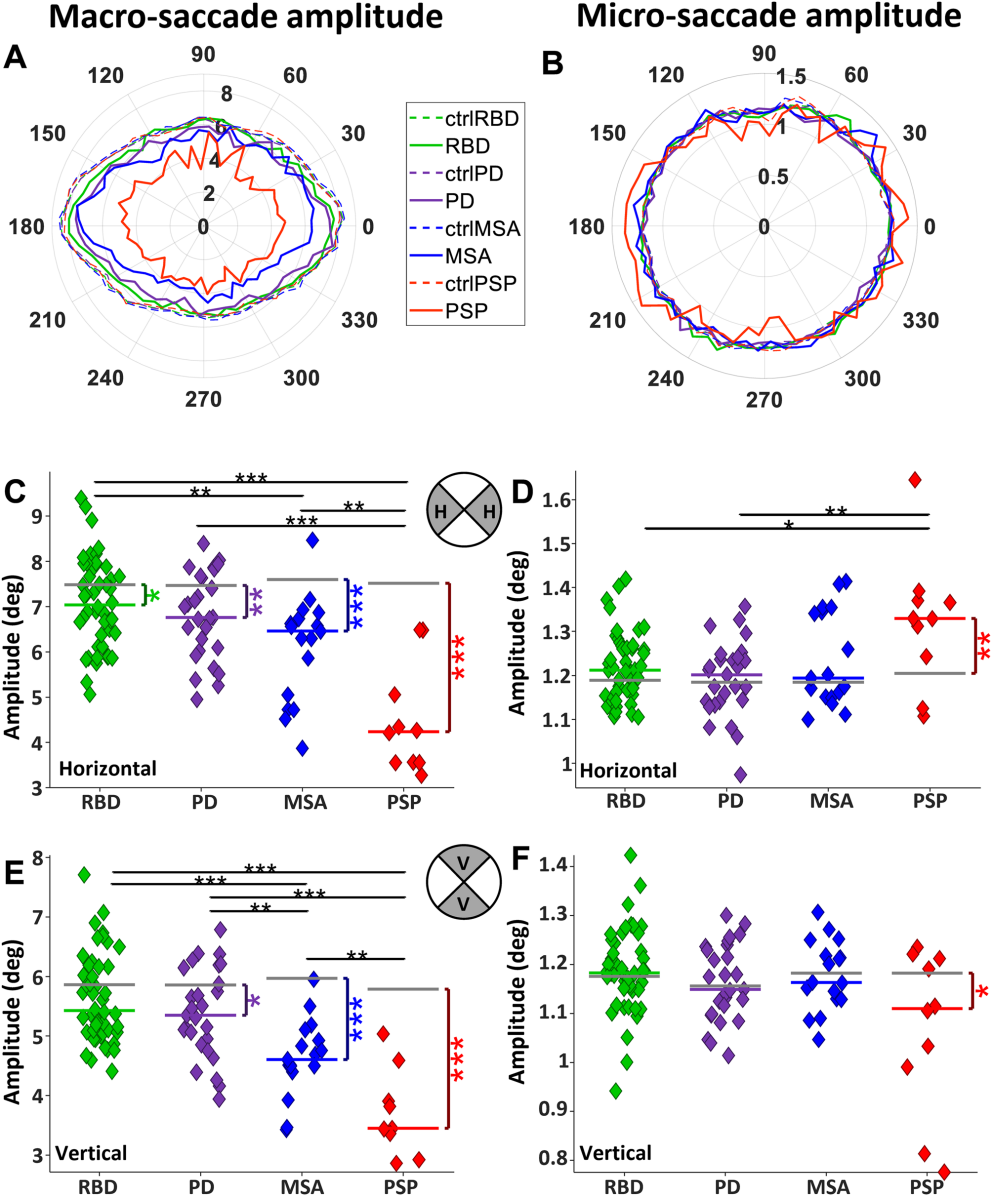
Characteristic of saccade amplitude in different directions. **A** Polar histogram of macro-saccade amplitude, **B** polar histogram of micro-saccade amplitude for each group. Polar coordinates are saccade directions, and each circle represents the average saccade amplitude within each group. The bin angle was 10 degrees. **C** and **D** Horizontal macro- and micro-saccade amplitude, respectively. **E** and **F** Vertical macro- and micro-saccade amplitude, respectively

We determined the average saccade amplitude for each of the 60 directions (Fig. 4A–B). PSP participants made the smallest macro-saccades amplitude in all directions, followed by MSA, then PD, and finally RBD, while CTRL made the largest macro-saccades (Fig. 4A).

Horizontal macro-saccade amplitude was reduced in all patient groups compared to CTRL (Fig. 4C; RBD: 7.04 saccades/s versus 7.49, *P* < 0.05, PD: 6.76 versus 7.46, *P* < 0.01, MSA: 6.46 versus 7.60, *P* < 0.0001, and PSP: 4.30 versus 7.52, *P* < 0.0001). RBD made larger macro-saccades than MSA (*P* < 0.01) and PSP (*P* < 0.0001). All αSYN groups made larger horizontal macro-saccades than PSP (*P* < 0.001). Horizontal micro-saccade amplitude was significantly larger in PSP versus CTRL (Fig. 4D; 1.33 degree versus 1.2, *P* < 0.01, all other comparisons of patients to CTRL were not significant (all *P* > 0.05)). PSP had a horizontal larger micro-saccade amplitude than RBD (*P* < 0.05) and PD (*P* < 0.01).

Vertical macro-saccades had reduced amplitude in PD, MSA, and PSP relative to CTRL (Fig. 4E; PD: 5.35 degree versus 5.86, *P* < 0.05, MSA: 4.60 versus 5.97, *P* < 0.0001, and PSP: 3.44 versus 5.82, *P* < 0.0001). Comparisons of αSYN groups showed that both RBD and PD had larger vertical macro-saccade amplitude than MSA (*P* < 0.001), while PSP had smaller vertical amplitude compared to all groups (*P* < 0.001). PSP had a smaller vertical micro-saccade amplitude than CTRL (1.11 versus 1.18, *P* < 0.05).

#### Saccade amplitude-velocity relationship

**Fig. 5:**
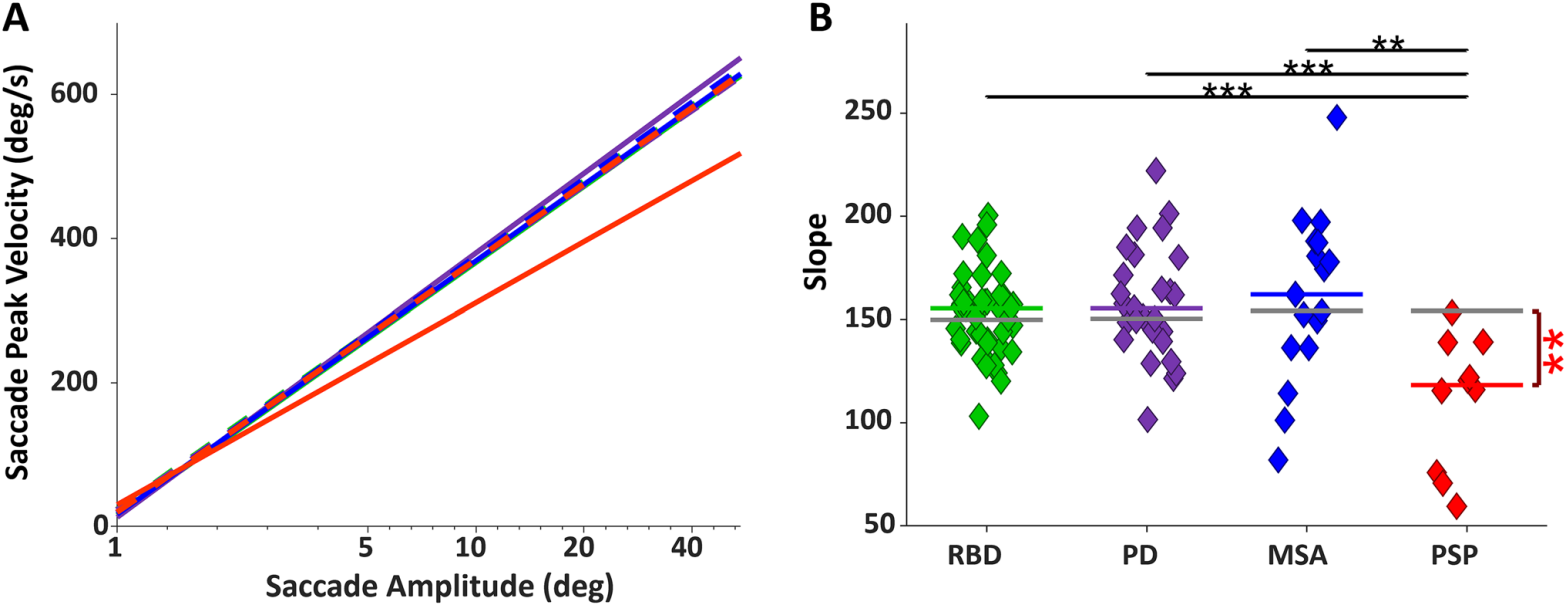
Main sequence. **A** Main sequence of all patient groups along with their matched CTRL. The X-axis is amplitude on a logarithmic scale. The linear fitting line is applied over all data points of the subjects in 10 different movies in all directions. **B** Slope of the fit line for the main sequence of each individual

The average main sequence (saccade amplitude vs. velocity [43, 47]; see Supplementary Fig. 3 for single subject fit) of all groups showed that PSP patients had significantly slower saccades than CTRL and all other patient groups (Fig. 5A). The slopes of the individual participants’ main sequence linear fits are shown in Fig. 5B. PSP had significantly slower saccades compared to CTRL (PSP: 118.37 degree/s versus 154.18, *P* < 0.01, all other comparisons of patients to CTRL were not significant (all *P* > 0.05)). PSP also had significantly slower saccades compared to RBD (*P* < 0.001), PD (*P* < 0.001), and MSA (*P* < 0.01).

### Analyses aligned on clip changes

#### Clip-aligned changes in saccade rate

**Fig. 6:**
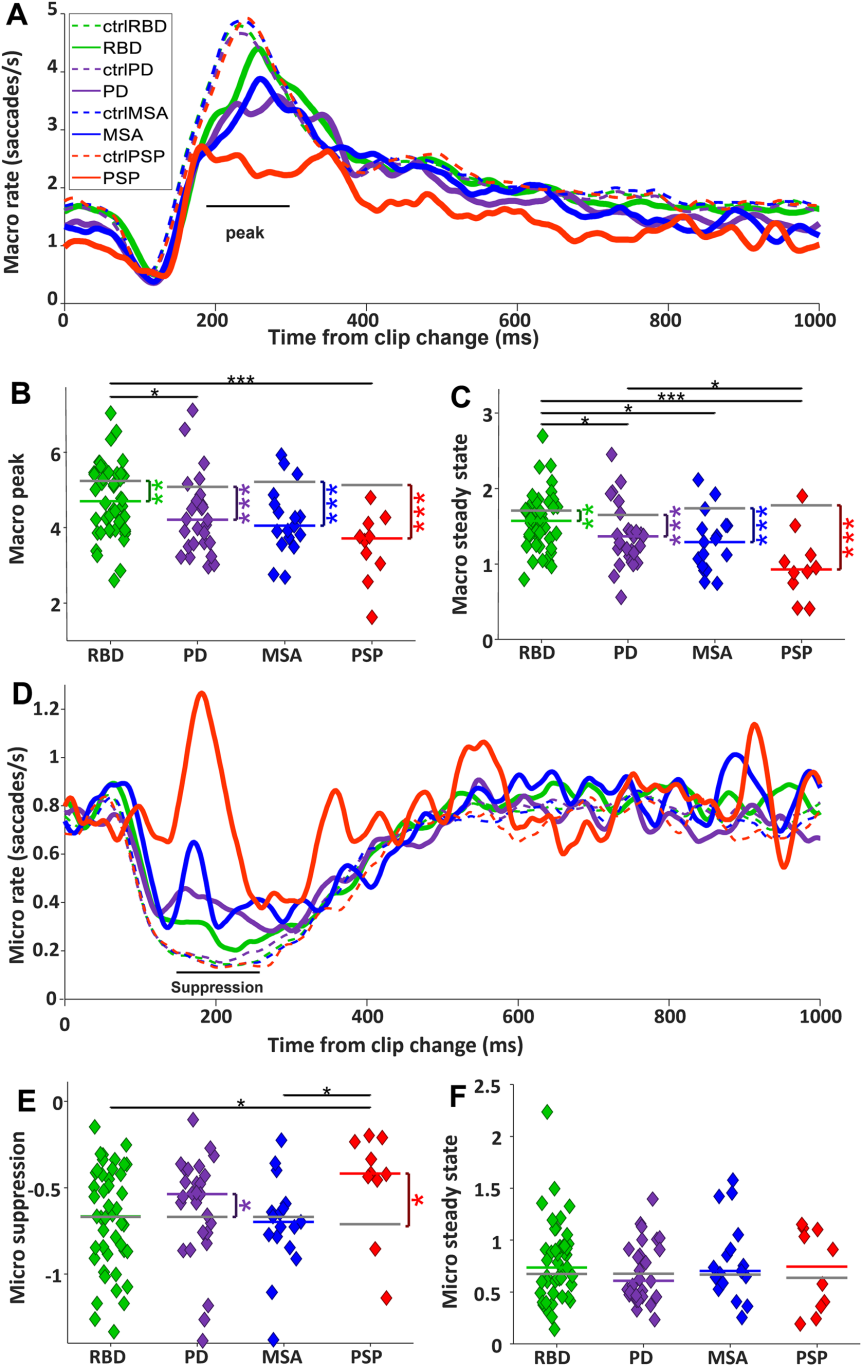
Saccade rate after clip change. **A** Macro-saccade rate after clip change. The black horizontal line shows the epoch in which the average macro-saccade peak was measured. Every trace represents the mean macro-saccades of all participants in all trials. **B** Median macro-saccade peak for each participant. **C** Median macro-saccade rate in steady state (1000–3000 ms after clip change). Notably, the most critical finding in panels B and C is the distinction between RBD and PD. **D** Micro-saccade rate after clip change. Every trace represents the mean micro-saccades of all participants in all trials. The black horizontal line shows the epoch in which the micro-saccade rate suppression has been measured. **E** Median of micro-saccade suppression magnitude. **F** Median micro-saccade rate in steady state

The clip transition represents a large perturbation in visual input to the brain. We examined the results of saccade and pupil responses that were influenced by these clip changes. About 65 ms after clip change, there was a momentary suppression in macro-saccade rate, followed by a rebound that started ~ 120 ms and peaked at approximately 200–250 ms (Fig. 6A). Finally, the saccade rate returned to a steady state rate about 400–500 ms after clip change. The baseline saccade rate prior to clip change was reduced in all patient groups (Supplementary Fig. 4A), but the depth of the suppression was not different across groups (Supplementary Fig. 4B). Most importantly, although the start of the saccade rebound (120–170 ms after clip change) was similar in patients and controls, the peak of the rebound was significantly reduced in all patient groups relative to controls (Fig. 6B; RBD: 4.70 saccades/s versus 5.23, *P* < 0.01, PD: 4.20 versus 5.08, *P* < 0.001, MSA: 4.05 versus 5.2, *P* < 0.001, and PSP: 3.70 versus 5.16, *P* < 0.0001). RBD had a higher saccade peak than PD (*P* < 0.05) and PSP (*P* < 0.001). Because the start of the rebound was relatively normal, we interpret that all subjects were motivated and attending to the task. The average saccade rate in the epoch 1000–3000 ms (steady state) after the clip changes was reduced in all patient groups relative to CTRL (Fig. 6C; RBD: 1.57 saccades/s versus 1.71, *P* < 0.01, PD: 1.36 versus 1.65, *P* < 0.0001, MSA: 1.29 versus 1.73, *P* < 0.0001, and PSP: 0.92 versus 1.79, *P* < 0.001). RBD had a higher steady state saccade rate compared to PD (*P* < 0.05), MSA (*P* < 0.05), and PSP (*P* < 0.001). PD also had a higher saccade rate compared to PSP (*P* < 0.05). When we separated the clips for high and low luminance, we did not observe differences in saccade rate based upon luminance levels of the clips.

Micro-saccade rate was also affected by the clip change (Fig. 6D). In CTRL, the micro-saccade rate dropped ~ 70 ms after clip change, and this suppression persisted until ~ 500 ms before returning to a steady state. The magnitude of suppression of micro-saccade rate was reduced in PD and PSP relative to CTRL (Fig. 6E; RBD: -0.67 saccades/s versus -0.67, *P* = 0.54, PD: -0.53 versus -0.67, *P* < 0.05, MSA: -0.70 versus -0.67, *P* = 0.90, and PSP: -0.42 versus -0.72, *P* < 0.05). RBD and MSA had larger suppressions than PSP (both *P* < 0.05). Steady state micro-saccade rate (1000–3000 ms after clip change) did not differ between the groups.

#### Clip-aligned changes in pupil size

**Fig. 7:**
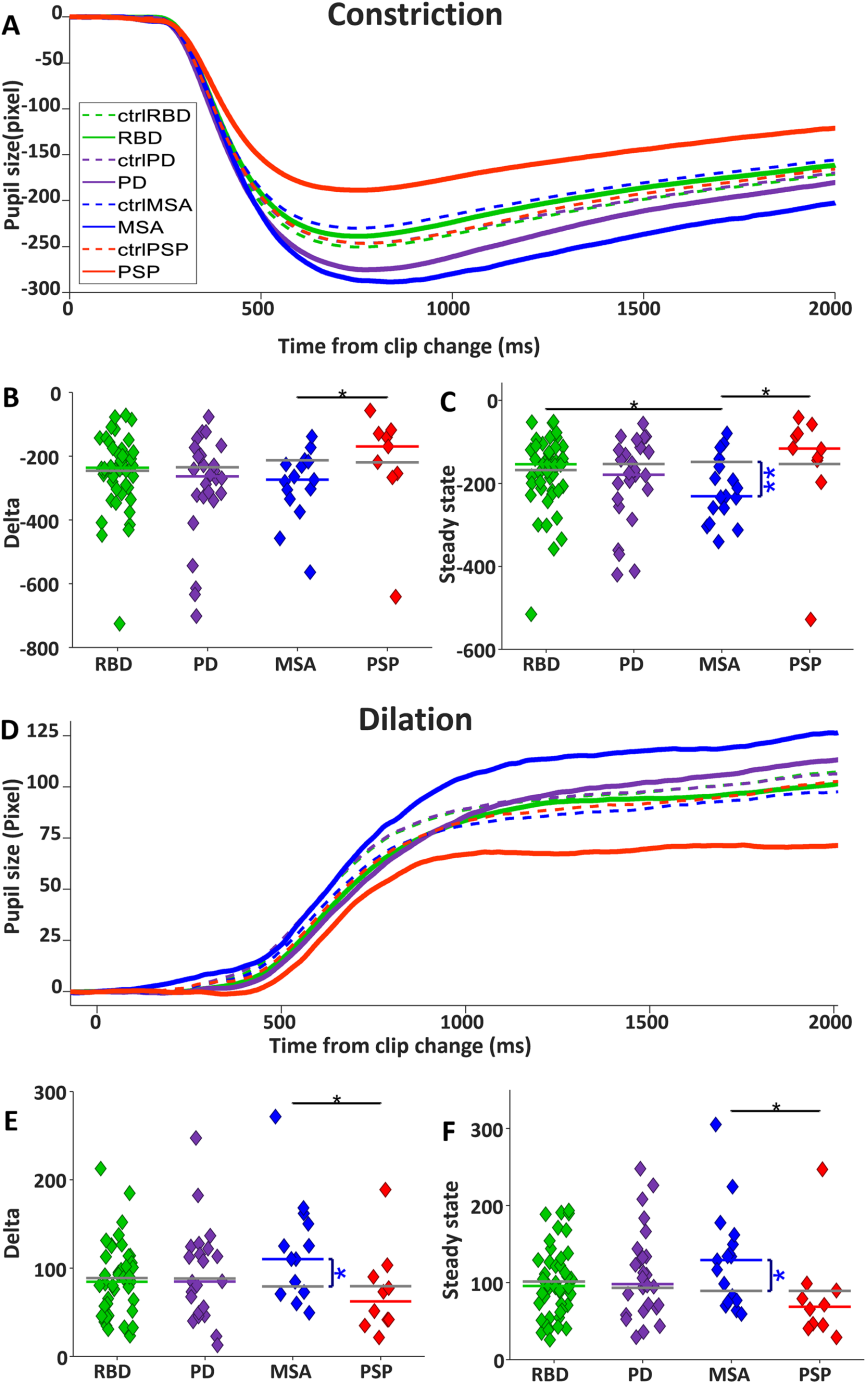
Pupil response. **A** Pupil constriction after clip change with positive luminance change. Time zero shows the onset of the clip change. **B** Median pupil constriction Delta and **C** median pupil size in steady state for each participant. **D** Pupil dilation after clip change with negative luminance change. **E** Median pupil dilation magnitude and **F** median pupil size in steady state

Changes in global luminance evoke transient pupil responses [48], and the clip changes included significant luminance changes on the screen that drive changes in pupil size. For the clip changes with the 20% most significant luminance increase (Fig. 7A), a robust constriction of the pupil was initiated ~ 300 ms after clip change and peaked at ~ 800 ms, followed by a gradual increase in pupil size over the next 2 s. The absolute pupil constriction change was smaller in PSP than CTRL but failed to reach a significance level (Fig. 7B; PSP:  – 169.21 pixels versus  – 217.73, *P* = 0.19). MSA and PD had a bigger pupil constriction delta than CTRL but failed to reach a significance level (PD:  – 262.9 pixels versus  – 235.26, *P* = 0.25, MSA:  – 273.96 versus  – 212.53, *P* = 0.06). RBD was very similar to CTRL in the size of pupil constriction delta (RBD:  – 236.53 pixels versus  – 245.25, *P* = 0.72). MSA had a significantly greater pupil constriction delta than PSP (*P* < 0.05). Relative pupil size in steady state following luminance increase (Fig. 7C) was more constricted in MSA relative to CTRL (RBD:  – 154,16 pixels versus  – 168.11, *P* = 0.80, PD:  – 179.92 versus  – 153.62, *P* = 0.53, MSA:  – 231,52 versus  – 148.86, *P* < 0.01, and PSP:  – 116.27 versus – 148.86, *P* = 0.11). In the steady state epoch, MSA had more constriction than RBD (*P* < 0.05) and PSP (*P* < 0.05).

For the clip changes with the 20% greatest decrease in global luminance, there was a robust dilation of the pupil that began ~ 400 ms after clip change, followed by an increase in pupil size until a steady state was reached at approximately 1000 ms (Fig. 7D). However, there were significant differences in the magnitude of this dilation response across groups. MSA had larger pupil dilation compared to CTRL, while PSP elicited smaller dilation than CTRL, but this was not significant (Fig. 7E, RBD: 84.59 pixels versus 88.72, *P* = 0.51, PD: 84.85 versus 88.31, *P* = 0.80, MSA: 110.36 versus 79.18, *P* < 0.05, and PSP: 62.44 versus 78.87, *P* = 0.25). Pupil dilation was larger in MSA than PSP (*P* < 0.05). Relative to CTRL, median pupil size after dilation in steady state was bigger in MSA (Fig. 7F, 129.09 pixels versus 89.23, *P* < 0.05), while it was smaller (not significant) in PSP (68.46 pixels versus 86.85, *P* = 0.10). RBD and PD displayed a similar pupil dilation with CTRL (RBD: 95.75 pixels versus 101.38, *P* = 0.91, PD: 97.75 versus 92.95, *P* = 0.64). MSA had larger pupil dilation in steady state than PSP (*P* < 0.05).

Some of these changes in the dynamics of pupil responses following luminance changes could be the result of different baseline pupil sizes in the different disorders. It is intriguing that pupil baseline size was elevated in MSA, but slightly reduced in PD and RBD (Supplementary Fig. 5). Baseline pupil size was greatly reduced in PSP, compared to CTRL and the αSYN groups.

### Correlations between oculomotor and clinical assessment

**Fig. 8:**
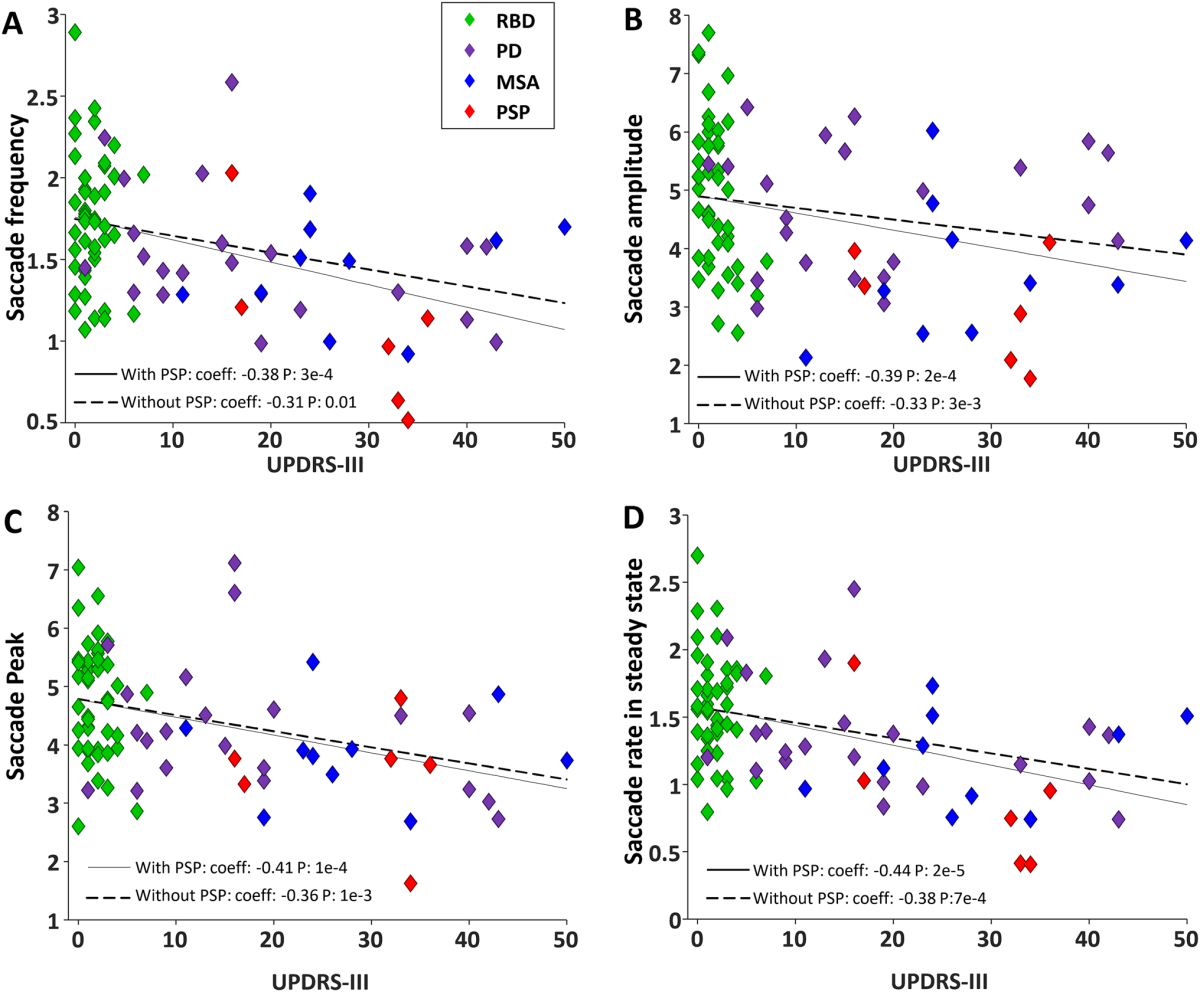
Relation between UPDRS-III and saccade. **A** Negative correlation between saccade frequency and UPDRS-III score. **B** Negative correlation between saccade amplitude and UPDRS-III score. **C** Negative correlation between saccade peak and UPDRS-III score. **D** Negative correlation between saccade rate in steady state and UPDRS-III. The solid and dashed black lines show the linear fit over data including PSP and without PSP, respectively

A correlation analysis with the UPDRS-III scores of all patients from all groups and their saccade (Fig. 8; Supplementary Fig. 7) and pupil (Supplementary Fig. 8) parameters was performed. We also repeated the analysis without including the PSP patients to isolate the correlations for the αSYN groups. Spearman correlation revealed that macro-saccade frequency was negatively associated with the severity of motor symptoms in the combined patient group (Fig. 8A; with PSP: ρ =  – 0.38, *P* < 0.001; without PSP: ρ =  – 0.31, *P* < 0.005). Saccade amplitude was also negatively correlated with UPDRS-III (Fig. 8B, with PSP: ρ =  – 0.39, *P* = 0.0002, without PSP: ρ =  – 0.33, *P* = 0.003). The rebound in saccade rate following the clip changes was negatively correlated to UPDRS-III score (Fig. 8C, with PSP: ρ =  – 0.41, *P* < 0.0001, without PSP: ρ =  – 0.36, *P* < 0.001), as well as the steady state saccade rate 1000–3000 ms after clip change (Fig. 8D, with PSP: ρ =  – 0.44, *P* < 0.001, without PSP: ρ =  – 0.37, *P* < 0.001). Neither micro-saccade rate (Supplementary Fig. 7A) nor micro-saccade suppression magnitude (Supplementary Fig. 7B) was correlated with UPDRS-III score, either with or without PSP included. We did not identify any significant correlations between pupil parameters and UPDRS-III scores (Supplementary Fig. 8A–D).

## Discussion

In this perspective exploratory study, we investigated parameters of oculo- and pupillomotor function in the manifest αSYN PD, MSA, and the prodromal αSYN RBD in comparison to the tauopathy PSP. We employed a Free Viewing paradigm (FV)—in combination with novel analysis methods of saccade and pupil behaviours- to study the above mentioned movement disorders. Previous studies have used visually guided saccade tasks to quantify horizontal and vertical gaze abnormalities [21, 22]. When uninstructed participants watched short video clips for only 10 min, this FV paradigm allowed us to answer the three questions lined out in the introduction as follows: (1) FV revealed qualitatively similar vertical gaze abnormalities as reported for the visually guided saccade task, but in addition, we describe several novel findings related to saccade and pupil behaviour as detailed below; (2) the behavioural results from FV differentiated between patients with αSYN and PSP –in principle in line with the results obtained with the visually guided saccade task; and (3) in the αSYN prodrome RBD, the FV paradigm allowed us to identify already discrete, but distinct saccadic abnormalities, which however are less pronounced than in PD and MSA patients.

### Saccade abnormalities in neurodegenerative movement disorders

All patient groups had altered saccade behaviour during the FV task, including increased centre bias (Fig. 1) and reduced saccade amplitude and frequency (Figs. 2, 3, 4). Thus, all patients with αSYNs or PSP – to varying degrees—harvested less visual information from the peripheral visual display, and instead focused their limited resources on the centre of the screen, which would greatly reduce their ability to process the whole gist of any clip.

The clip transitions had a profound impact on saccade production (Fig. 6). Within ~ 70 ms of clip transition, the macro-saccade rate plunged to a nadir ~ 120 ms before rebounding. This initial suppression in saccade rate was the result of large changes in the visual display at clip change [45, 49] and was likely produced by visual input passing through the superior colliculus (SC) to the brainstem omnipause neurons (OPNs) [50] which gate all saccades via direct inhibition of premotor excitatory and inhibitory burst neurons [51–53] in the paramedian pontine reticular formation (PPRF) and the rostral interstitial nucleus of the medial longitudinal fasciculus (riMLF). OPNs have transient visual responses [54–56] and so the visual perturbation produced by the clip change, which is known to activate neurons in the SC [34], likely led to an increase in OPN discharge which would immediately inhibit saccade burst neurons in the riMLF and PPRF and lead to saccade suppression.

In structured oculomotor tasks, visually triggered saccades are typically initiated more than 90 ms after target appearance and can be further characterized as express saccades or regular latency saccades [32, 57]. Saccades with reaction times < 90 ms are not visually triggered [33, 58]. Analogous to the structured pro-saccade task, in FV, saccade triggered < 90 ms after clip change preceded the transient epoch of saccade suppression, and the ensuing rebound in saccade rate represents the shortest latency visually triggered saccades, which could include both express (90–140 ms) and regular (> 140 ms) latency saccades. Express saccades, the shortest latency visually triggered saccades that human can make [57], are produced when transient visual signals travelling through the SC become the saccade command [59, 60].

Following the clip transitions in the FV task, the depth of the saccade suppression and initial part of rebound was intact in all patient groups. However, the peak of the saccade rebound was significantly blunted in all patient groups (Fig. 6B), which is analogous to the time of regular latency saccades in the pro and anti-saccade tasks (SRT > 140 ms) [32]. Because the initial part of the saccade rebound was intact, we interpret this to mean that all participants were motivated and attended to the task. The reduced frequency of saccades at this time was likely the result of cognitive impairments due to neurodegeneration in cortical/basal ganglia circuits affecting or delaying key inputs to the SC [21, 23, 30, 61]. This observation which is analogous to increased latency of correct saccades among PD patients performing the anti-saccade task. In contrast, the generation of automatic visually triggered pro-saccades remained relatively unimpaired in PD [23, 30], likely because these automatic saccades are driven by visual inputs from occipital and parietal cortex to the SC, regions of the brain that are less impacted in the diseases studied here.

The FV task provided an assessment of many saccade parameters. However, we were not able to determine subtle saccade abnormalities related to dysmetria because we did not define visual targets in the video clips. The visually guided saccade task is ideal to investigate saccade dysmetria and the difference between vertical and horizontal saccades. The FV task is better for measuring ongoing and continuous saccade and micro-saccade behaviour, and pupil behaviour without having to introduce any complex instructions or task parameters.

The SC represents a competition map for the generation of saccades in a winner take all manner [62] in which only one spatial location can issue a saccade burst at any one time. Likely due to the reduced macro-saccade rate following the clip change (Fig. 6A, B), the micro-saccade rate was less suppressed following clip change in PD and PSP (Fig. 6D, E). However, the micro-saccade steady state was not increased in the patient groups (Fig. 6F), despite the significant reductions in macro-saccade steady state in all patient groups (Fig. 2C). So this inverse relation between macro- and micro-saccade rates was not consistent across the entire clip but was most evident immediately following clip transition (< 500 ms).

Other brain disorders, such as the psychiatric disorder schizophrenia, have also been studied in terms of eye movement dysfunctions. According to a recent study, patients with schizophrenia showed fewer fixations with longer duration and smaller and lower saccades during a free visual exploration compared to CTRL [63]. Silberg et al. also showed that when patients with schizophrenia explore movies of real-life scenes, they had a strong centre bias behaviour and their gaze was independent of saliency based features of the movie [64, 65]. Schizophrenic individuals explored a smaller area of the visual scene compared to CTRL [65]. This pattern of results is similar to what we observed in all of our patient groups and may be indicative of general frontal cortex dysfunction. Whether this is a genuine feature of schizophrenia or due to antidopaminergic therapy needs to be clarified.

### Vertical saccade deficits in neurodegeneration

All patients had a significant reduction in vertical saccade rate which was greatest in PSP (Fig. 3E). PD patients make hypometric saccades in vertical and horizontal directions [66, 67], but do not exhibit downward vertical gaze paresis, which is typical in PSP [18, 20, 39]. This dramatic vertical gaze palsy in PSP is likely the result of degeneration in the midbrain that impacted the riMLF. This structure houses the vertical saccade burst neurons that project directly to the pools of vertical extraocular muscle motoneurons in the oculomotor and trochlear nuclei [68]. Reduction in signals from these burst neurons in the riMLF will make it harder to initiate the vertical component of saccades, and those saccades will have a reduced amplitude and velocity. This is the pattern we observed in PSP, where it appears that these neurons were selectively damaged, leading to vertical gaze palsy. This hypothesis is supported by structural abnormalities in PSP that are known to often impact the midbrain and hence riMLF [69], which may appear small and pathologic [13].

### Pupil characteristics in neurodegeneration—opposite effects in PSP versus MSA

Pupil responses were abnormal in the different patient groups, but in dramatically different ways for the PSP versus the MSA group (Fig. 7) which suggest very different actions of pathophysiology. All participants showed a very robust centre bias (Fig. 1), and pupil size is determined by global luminance. Therefore, the pupil differences we described cannot be attributed to local luminance differences based upon the location of fixation. Across the duration of the free viewing of video, pupil size for the PSP group was significantly smaller than for the MSA group (Supplementary Fig. 5). Following clip transition to darker or brighter clips, pupil dilation and constriction responses were attenuated in PSP but exaggerated in MSA (Fig. 7). Despite these large differences in the magnitude of the pupil responses between PSP and MSA, there were no differences in the onset latency of the constriction or dilation responses (Supplementary Fig. 6), suggesting that the deficits likely arise from central (i.e., brainstem) rather than peripheral (i.e., retinal), origin.

A number of factors influence pupil size in addition to luminance, such as cognitive and emotional factors, sensory saliency, and arousal [70]. The dominant luminance pathway consists of retinal input to the pretectal olivary nuclei via intrinsically photosensitive retinal ganglion cells [71]. Neurons in the pretectal olivary nucleus project directly to the Edinger Westphal nucleus (EW) [72, 73]. Many different brainstem nuclei and pathways are responsible for the non-luminance modulations of pupil size [74]. The locus coeruleus (LC) in the pons is a key structure in pupil control [75]. The discharge of LC neurons is correlated to the slow changes in pupil size that are related to arousal [75]. More recently, another non-luminance pathway has been identified through the SC [74]. The same SC neurons that project to riMLF and PPRF also collateralize into regions of the central mesencephalic reticular formation (cMRF) [76, 77], which then projects to EW [78] to influence pupil size. As a result, cognitive control signals from cortex that flow through the SC have a route to influence pupil size.

Pathophysiology of the LC has been implicated in the early stages of PD, typically at the prodromal stage II of Braak and coworkers [79]. Thus, alterations in LC activity, which likely occur in αSYN, would lead to altered pupil control. Consistent with our findings, previous studies have also identified exaggerated pupil responses in αSYN, including larger pupil diameter after light adaptation in PD [80], larger pupil size after both light and dark adaptation in MSA [81]. However, other studies have identified conflicting results regarding pupil dysregulation in αSYN, including finding similarities in pupil baseline between PD and CTRL [82], reduced constriction amplitudes in PD, and longer latency of the light reflex [80, 82]. However, we observed no differences in constriction or dilation latency (Supplementary Fig. 6). PD patients have an autonomic imbalance and are more sensitive to light [27, 83–85]. Previous studies have also identified additional abnormal pupil behaviour in MSA; for instance, they lack a bigger pupil response to stress [86, 87], the average constriction and dilation velocities were considerably slower than controls [11], and larger pupil size after both light and dark adaptation in MSA [81]. The above conflicting findings are likely the result of different stimulus manipulations on the retina. The pupil responses that we observed in the FV task involved stimulation of much of the retina. Additional research will be necessary to determine what is the optimal visual stimulus required to reveal consistent pupil deficits in these patient groups.

Part of the hypothesis of the spread of pathophysiology in αSYN includes early involvement of the LC [79], which plays a critical role in regulating pupil size concerning arousal [75]. It has been shown in monkeys that LC discharge is tightly correlated to pupil size; greater discharge leads to increases in pupil size, and microstimulation of LC also increases pupil size [88]. It is hard to reconcile how the loss of neurons in LC leads to increased pupil size in αSYN.

PSP is known to have pathophysiology in the midbrain that may impact EW and cMRF, which are near riMLF [13, 14]. Therefore, midbrain pathophysiology may impact either neurons within EW or afferents to this nucleus in the midbrain. EW receives both excitatory and inhibitory connections from the cMRF and could conceivably produce the opposite pupil effects we observed in PSP versus MSA (Fig. 7).

### Discrete saccadic abnormalities in RBD are pronounced in PD and MSA

We specifically included the isolated RBD patient group in our study to determine whether this prodromal αSYN group started to reveal patterns of abnormality identified in PD and MSA. Although centre bias was exaggerated in PD and MSA, RBD was similar to CTRL (Fig. 1). RBD made less macro-saccades than CTRL, but more than PD and MSA (Fig. 2A, 6A–C). All patient groups made smaller macro-saccades than CTRL, but this effect was very modest in RBD and much stronger in PD and MSA (Fig. 3A, C, E). Pupil responses in RBD were not predictive of changes in PD and MSA. These results reveal that RBD patients already display some saccade control deficits (macro-saccade frequency and amplitude) which are intensified in PD and MSA. Our results suggest that saccade parameters were already changing in RBD, but pupil responses were not. These altered saccade responses in RBD might represent early markers of αSYN. However, long-term studies, particularly including subjects who phenoconvert from RBD to PD or MSA during the study, are needed to confirm these findings.

Other studies have tried to identify early abnormalities in oculo-pupillo-motor function in the prodromal RBD condition [6, 21, 22, 89, 90] that could be used as indicators for early diagnosis of αSYN. Perkins et al. [21] identified attenuated pupil responses for RBD and PD patients performing an interleaved pro and anti-saccade response following the appearance of a central fixation spot, but this visual stimulus was a tiny spot confined to the fovea. In our study, the clip change was a substantial visual stimulus, covering the entire screen in front of the participant that presumably activated most of the retina. In this situation, RBD and PD pupil responses were not different from CTRL, however MSA had exaggerated responses that were significant for dilation (Fig. 7E). Additional research is required to identify whether retinal disturbances contribute to the pupil abnormalities we have reported in one, but not in the other αSYN disorders and whether these disturbances are uniform across the retina or are confined to specific regions of the retina (e.g., fovea vs. extrafoveal).

### Linking eye tracking to UPDRS-III

The UPDRS is part of the standard for diagnosis of PD [91]. We found that saccade frequency, average saccade amplitude, and the magnitude of the rebound burst of saccades after the clip change were all negatively correlated to motor function, assessed with the UPDRS-III (Fig. 8). Other studies have also identified saccade parameters that correlated with clinical scores [92–94]. None of our pupil measures were correlated to UPDRS-III (Supplementary Fig. 8). Pupil assessment is not part of UPDRS-III [95] but may provide some unique measures that may be altered in αSYN, at least for MSA. Pupil measures may also be sensitive for distinguishing PSP from PD and MSA. Our results suggest that pupillometry may tap into additional brainstem circuits and provide additional measures of dysfunction.

## Conclusions

We used a simple FV paradigm to identify oculo-pupillo-motor abnormalities in various neurodegenerative movement disorders. We identified potential prodromal biomarkers in RBD and differences between αSYN and the tauopathy PSP, suggesting that the FV task may be a tool to identify prodromal αSYN and help to distinguish early manifest αSYN from early PSP. Future intra-individual follow-up studies are required in RBD patients to determine whether the so far observed subtle changes in oculo-pupillo-motor measures will progressively increase over time and allow the prediction of the phenoconversion of RBD into manifest αSYN. These longitudinal studies will show whether oculo-pupillo-motor parameters can reliably classify neurodegenerative movement disorders in the manifest stage, and even more challenging, during their prodromal progression towards phenoconversion.

## Supplementary Information

Below is the link to the electronic supplementary material.

Supplementary file1 (DOCX 37 KB)

Supplementary file2 (PNG 186 KB)

Supplementary file3 (PNG 614 KB)

Supplementary file4 (PNG 168 KB)

Supplementary file5 (PNG 1010 KB)

Supplementary file6 (PNG 1556 KB)

Supplementary file7 (PNG 212 KB)

Supplementary file8 (PNG 466 KB)

Supplementary file9 (PNG 730 KB)

Supplementary file10 (PNG 193 KB)

## Data Availability

The original data will be available upon request.

## Abbreviations

αSYN: Alpha-synucleinopathy
BDI: Beck's depression inventory
cMRF: Central mesencephalic reticular formation
EW: Edinger westphal nucleus
H&Y: Hoehn and Yahr scale
LC: Locus coeruleus
MoCA: Montreal cognitive assessment
MSA: Multiple system atrophy
OPN: Omnipause neurons
PD: Parkinson’s disease
PDNMS: PD-non-motor-symptom scale
PPRF: Paramedian pontine reticular formation
PSP: Progressive supranuclear palsy
REM: Rapid eye movement
RBD: Isolated REM sleep behaviour disorder
RBDSQ: REM sleep behaviour disorder screening questionnaire
riMLF: Rostral interstitial nucleus of the medial longitudinal fasciculus
SC: Superior colliculus
UPDRS: Unified Parkinson’s disease rating scale
