# Supplementary material for: Eye tracking identifies biomarkers in α-synucleinopathies versus progressive supranuclear palsy
Source: J Neurol. 2022 Apr 30;269(9):4920–38. doi: 10.1007/s00415-022-11136-5 (PMC9363304; doi:10.1007/s00415-022-11136-5)
Supplement: Supplementary file 1 — Supplementary file1 (DOCX 37 KB) [file 415_2022_11136_MOESM1_ESM.docx]

**Eye tracking identifies biomarkers in α-synucleinopathies versus progressive supranuclear palsy**

Mahboubeh Habibi,^1,2^ Wolfgang H Oertel,^1^ Brian J White,^2^ Donald C Brien,^2^ Brian C Coe,^2^ Heidi C Riek,^2^ Julia Perkins,^2^ Rachel Yep,^2^ Laurent Itti,^4^ Lars Timmermann,^1^ Elisabeth Sittig,^1^ Christoph Best,^1^ Annette Janzen ^1,†^ and Douglas P. Munoz ^2,3†^

**^†^** **shared senior authorship.**

**Author affiliations:**

1 Department of Neurology, Philipps-University, 35043 Marburg, Germany

2 Centre for Neuroscience Studies, Queen’s University, Kingston, Ontario, Canada

3 Department of Biomedical and Molecular Sciences, Queen’s University, Kingston, Ontario, Canada.

4 Department of Computer Science, University of Southern California, Los Angeles, CA 90089

Correspondence to: Douglas Munoz, Mahboubeh Habibi

Full address: Dr. Douglas P. Munoz, Centre for Neuroscience Studies 18 Stuart Street, Kingston ON K7M 4A4.

E-mail: doug.munoz@queensu.ca, Mahbubeh.hb@gmail.com

**Supplementary Materials**

**Recording and Apparatus**

A video-based monocular eye tracker was used to monitor eye position, pupil size, and blink rate at a rate of 500 Hz (Eyelink-1000 Plus, SR Research Ltd, Osgoode, ON, Canada). Stimuli were shown on a 17-inch LCD panel (1280x1024 pixels, 32-bit color, 60 Hz refresh rate), corresponding to a viewing angle of 32x26° controlled by the operator through a Dell Latitude E7440 Laptop. Videos were delivered at 30 fps using custom software in Ubuntu 13 to interface with the eye tracker via the SR Research API. The distance between the eyes and the monitor and infrared camera was adjusted at 60 cm, the optimum distance between the camera and the eye. All recordings and calibrations were conducted monocularly, using the right eye as the reference point. To begin, a nine-point grid was used to calibrate the eye location (eight around the periphery and one central). The stimuli were flashed in random patterns across the screen, and the participant was required to focus on each one until the next appeared. Following calibration, the procedure was repeated to ensure that the average error between fixation and stimulus was less than 1° and that there was no loss of eye tracking. To verify that observed substantial variations were not attributable to differences in location, both video-based eye-tracking devices were subjected to rigorous testing on a regular and recurring basis to assure consistency across machines. This study used a spectrometer to ensure that the eye trackers’ displays emitted an identical amount of luminance, which had no impact on pupil baseline, constriction, or dilation levels.

## **Participants**

**Exclusion criteria:**

All patients recruited were 45 - 84 years of age. We did not recruit the following patients: 1) patients with a secondary RBD or parkinsonian syndrome (e.g., drug-induced, subcortical arteriosclerotic encephalopathy); 2) patients who were taking medications that may alter pupillary responses (e.g., anticholinergics, benzodiazepines, beta receptor blockers, pilocarpine, or other drugs if indicated in the manufacturer’s information); and 3) patients with glaucoma, pronounced strabismus, or uncorrected refractive error> ± 5 diopters.

Each cohort had a maximum possible number of participants measured. The size of each group was mostly determined by the disease’s epidemiology and also the number of patients who had attended Marburg’s University Hospital and could therefore be recruited.

Following data collection, all data was processed through a fully automated pipeline, where various objective filters and criteria were applied regardless of the participant’s characteristics. Patients with poor data quality were excluded from these analyses. Besides that, each individual who did not meet the requirements (e.g., additional blinks during a specific time frame) has been removed for each particular variable.

There were reductions to the patient group sizes listed below for specific variable analyses. All other analyses were conducted with the full complement of participants. In the centre bias analysis (Fig. 1), there were 8 PSP participants used for analysis. There were 9 PSP participants used for the analysis of vertical saccade amplitude. For the analysis of pupil constriction delta, there were 26 PD, 15 MSA, 9 PSP, and 109 ctrlPD. For the analysis of pupil dilation delta there were 34 RBD, 22 PD, 13 MSA, 93 ctrlRBD, 91 ctrlPD, 65 ctrlMSA, and 30 ctrlPSP. Each figure description in the following sections includes the list of further included subjects.

**Supplementary Fig. 1** Age distribution of all patients (filled circles) and their respective age-matched controls (empty circles). Thick and thin vertical lines represent the median and mean age for each group, respectively. The CTRL cohort was collected in Kingston, Canada, and consisted of 132 individuals. To control for age effects, each patient group was matched with all CTRL participants with a maximum age difference of one year from each patient in that group. This resulted in 4 CTRL groups: one for each patient group. Supplementary Table 1 provides additional details from each patient and control group.

**Supplementary Fig. 2** **Experiment paradigm.** **(A)** Illustration of all movie trials. Every movie consisted of ~17 different scenes (clips), which lasted ~60 s in total to produce 10 movies in total(vertical boundary black lines). The timing of vertical black lines was used to match clip-based Saccade rate evaluations(next panel). We computed the luminance changes at each clip change that impacted pupil size. We defined “delta” as the change in luminance between the current frame and the previous frame. We then selected the top 20% of positive luminance deltas (clips with the greatest increases in luminance; green vertical lines) and the top 20% negative deltas (clips with the greatest decreases in luminance; red vertical lines). This resulted in 30 positive delta clips and 30 negative delta clips which were used to analyze pupil constriction and dilation responses. **(B)** Panel B depicts several data analyses performed on each clip. We regarded each clip as a separate trial and then averaged the saccade and pupil responses across trials to obtain an average saccade and pupil response for each participant and group (e.g., Figs. 6 and 7, respectively). The black line depicts the macro-saccade rate following a clip change (all rectangles in panel A), and the blue line depicts the micro-saccade rate at the same epochs. The red curve shows the pupil’s dilation in the clip changes with luminance decrease. The pupil reacts with dilation until it reaches a steady state. The green curve shows pupil constriction after clip changes with luminance increase. In these trials, the pupil starts to constrict, followed by dilation, and ends up in a steady state. Time zero shows the start of the clip change. The time at which the steady state response was computed is indicated by the shaded region. Other descriptions on the image pertain to the times when these parameters were collected.

**Saccade and pupil analyses**

We automated all saccade detection. We computed the z-score of the velocity-amplitude relationship (main-sequence [1]) for each eye movement that was initially coded as a saccade to distinguish non-physiological data from real saccades. Specifically, initially coded saccades whose z-score was > ±3 SD were considered outside the range of a normal saccade and were removed. This resulted in the removal of 4% of the initially detected saccades. We then defined macro-saccades as all saccades ≥ 2° amplitude and micro-saccades [2–7] as all saccades < 2° amplitude.

We defined any period between successive saccades as a fixation period and quantified the fixation duration. Fixation durations < 50 ms were excluded because they have been shown to not activate the fixation system in the brainstem [8]. The coordinates of each fixation were used to create gaze distribution maps. We created a 2D histogram with 32 x 26 bins (bin size: 1° of visual angle) of all fixations within a given movie. We then applied a Gaussian smoothing function (SD = 0.5 pixels) to the resulting image, which produced an average heatmap of the probability of gaze for each participant across all 10 movies. We also calculated the difference in gaze distribution for each patient group and its respective control group to generate difference gaze probability maps. To summarize these difference probability maps, we extracted the data along the horizontal and vertical meridian (an averaged ±5º strip across the meridian) of the difference maps to produce 2D line plots to illustrate the differences better. Lastly, centre bias [9] was calculated for each participant and was defined as the mean ±5º around the centre of the probability map for each participant.

**Clip-aligned analyses**

In the clip-aligned analyses, we computed the macro- and micro-saccade rate (saccades / s) for each participant using a peri-stimulus time histogram (PSTH, 2 ms bin width due to the 500 Hz sample rate). We then smoothed these PSTH traces using a MATLAB smoothing function (local regression using weighted linear least squares and a 1st degree polynomial model with a 50 sample span). For each participant, we extracted various parameters from these curves. The smoothing served to reduce the probability of false alarms in detecting meaningful dips and peaks in the curves and was verified by stepping through each participant’s data and observing the detected parameters. For the macro-saccade rate curves, we computed a baseline rate for a given participant, which was the average rate in the epoch from -200 to +50 ms relative to the clip change. We computed the magnitude and timing of the dip in macro-saccade rate (“saccade suppression” [10]) following the clip change. Specifically, macro-saccade suppression was defined as the difference between the baseline rate and the minimum rate within an epoch from 70-200 ms post clip change. We computed the peak macro-saccade rate as the maximum value within an epoch from the time of suppression (described above) to 300 ms post clip change. Following this transient peak of saccades, the rate returns to a steady state, and the average saccade rate during the steady state was reported, which was the average from 1000-3000 ms after clip change.

We extracted various parameters from the clip-aligned pupil responses from the negative and positive luminance delta clip changes. For each participant, we smoothed each of the 30 zero-normalized clip-aligned pupil traces using a MATLAB smoothing function (local regression using weighted linear least squares and a 1st degree polynomial model with a 50 sample span). We then took the first derivative of the smoothed traces (pupil velocity) and applied the smoothing function again, because the derivative amplifies any remaining noise and would otherwise result in false alarms in detecting reliable parameters from the traces. We determined the point where the velocity curves were significantly different from a baseline velocity (epoch ±100 ms relative to the clip change) using a running signed-rank test (1-tailed; i.e., negative velocity in the constriction condition, and positive velocity in the dilation condition). Constriction/dilation latency for each participant was taken as the point where the curves were significantly different from baseline for at least 10 consecutive samples within an epoch from 100-500 ms post clip change in the constriction condition, and 200-600 ms post clip change in the dilation condition (to account for the slower dilation response). We also extracted the point where the velocity curves again became not significantly different from the baseline, and the difference in pupil size between that point and baseline was taken as the delta. Peak velocity for each participant was the maximum velocity in an epoch starting from the constriction/dilation latency (described above) for 200 ms. The time of peak velocity was also extracted. Finally, a steady state pupil parameter was extracted and was defined as the pupil size from 1000-3000 ms post clip change.

**Supplementary Fig. 3** Main sequence. The main sequence of saccade peak velocity against amplitude in one representative CTRL subject. The X-axis is amplitude on a logarithmic scale. The linear fit line is applied over all macro-saccades of the subject across 10 movies.

**Supplementary Fig. 4** Saccade rate after clip change. **(A)** Saccade rate baseline. The average saccade rate in the epoch from -200 to +50 ms relative to the clip change **(B)** Saccade suppression following clip change. The minimum saccade rate within an epoch from 70-200 ms post clip change.

**Pupil sensitivity to luminance change (Supplementary Fig. 5).**

Screen luminance changes drive the pupil to react, and it has a negative correlation with pupil size. To that purpose, we assessed all luminance changes in clip transitions to see how they affect pupil response, and we reported the mean pupil size of the entire group for each clip change (Supplementary Fig. 5A). Fitting a line across the data points reveals that pupil size changes as luminance increases. MSA was higher than all other groups, and PSP was the lowest. The fitted line characteristics for each participant (not shown) indicated that RBD and PSP had considerably lower median Y-intercepts than CTRL, particularly PSP ranking lower than all other patient groups (Supplementary Fig. 5B). The median slope of the lines was lower in PSP than in CTRL (Supplementary Fig. 5C). Altered group sizes were 34 RBD, 23 PD, 16 MSA, 8 PSP, and 108 ctrlPD.

**Pupil response (Supplementary Fig. 6).**

In the clip changes with luminance increase, there is a pupil constriction which happens after about 230 ms (Supplementary Fig. 6A). Altered group sizes were 26 PD, 15 MSA, 9 PSP, and 109ctrlPD. Constriction latency was not different between groups. Pupil dilation latency was about 400 ms and did not differ between groups (Supplementary Fig. 6B). Altered group sizes were 34 RBD, 22 PD, 13 MSA, 93 ctrlRBD, 91ctrlPD, 65 ctrlMSA, and 30 ctrlPSP.

**Correlations between oculomotor and Clinical assessment (Supplementary Figs. 7,8).**

All patients underwent the Unified Parkinson Rating Scale III (UPDRS scores III) and/or Movement Disorder Society (MDS)-UPDRS scales III as part of their clinical routine. We converted all MDS-UPDRS III scores to UPDRS-III scores for consistency. We utilized the formula proposed by Goetz et al. for this purpose [11]. On the same day as eye movement assessment, UPDRS-III and all other clinical data were collected.

We investigated the relationship between UPDRS-III and macro-saccade rate and discovered that there was a negative relationship between UPDRS-III score and macro-saccade frequency (Fig. 8A), macro-saccade amplitude (Fig. 8B), macro-saccade peak (Fig. 8C), and macro-saccade rate in steady state (Fig. 8D). We then examined the correlation between UPDRS-III and micro-saccades (Supplementary Fig. 7). There was no correlation between micro-saccade frequency (Supplementary Fig. 7A) and micro-saccade suppression magnitude after clip change (Supplementary Fig. 7B) with UPDRS.

There was no connection between pupil dilation delta and steady state with UPDRS-III score (Supplementary Fig. 8A-B). Furthermore, no association was seen between pupil constriction delta and steady state using UPDRS-III (Supplementary Fig. 8C-D).

**Limitations and strength**

PD patients were pooled into one cohort, thus we did not differentiate between untreated PD, PD under treatment, and PD patients in defined OFF treatment. Future work will be required to determine if patients in these different subgroups have different responses to Free Viewing. The sample size of the MSA and PSP patient groups was rather small when compared to the sample size of RBD and also PD patient groups. This is due to the relative scarcity of patients with MSA and PSP, relative to the abundance of patients with RBD and PD. There were no biases introduced throughout the data analysis because the data were completely processed automatically.

This method has been used to a variety of neurological and psychiatric disorders in ongoing projects of our lab, and the next step is to compare different neuropsychiatric disorders together.

**References**

1. Bahill AT, Clark MR, Stark L (1975) The main sequence, a tool for studying human eye movements. Mathematical biosciences 24:191–204

2. Martinez-Conde S, Macknik SL, Hubel DH (2000) Microsaccadic eye movements and firing of single cells in the striate cortex of macaque monkeys. Nature neuroscience 3:251–258

3. Martinez-Conde S, Macknik SL, Troncoso XG, Dyar TA (2006) Microsaccades counteract visual fading during fixation. Neuron 49:297–305

4. Otero-Millan J, Schneider R, Leigh RJ, Macknik SL, Martinez-Conde S (2013) Saccades during attempted fixation in parkinsonian disorders and recessive ataxia: from microsaccades to square-wave jerks. PLoS One 8:e58535

5. Otero-Millan J, Serra A, Leigh RJ, Troncoso XG, Macknik SL, Martinez-Conde S (2011) Distinctive features of saccadic intrusions and microsaccades in progressive supranuclear palsy. Journal of Neuroscience 31:4379–4387

6. Alexander RG, Macknik SL, Martinez-Conde S (2019) Microsaccades in applied environments: Real-world applications of fixational eye movement measurements. Journal of Eye Movement Research (6). https://doi.org/10.16910/jemr.12.6.15

7. Susana Martinez-Conde, Stephen L Macknik, David H Hubel (2004) The role of fixational eye movements in visual perception. Nature reviews neuroscience 5:229–240

8. Bergeron A, Guitton D (2001) The superior colliculus and its control of fixation behavior via projections to brainstem omnipause neurons. In: Progress in brain research. Elsevier, pp 97–107

9. Tseng P-H, Carmi R, Cameron IG, Munoz DP, Itti L (2009) Quantifying center bias of observers in free viewing of dynamic natural scenes. Journal of vision 9:4–4

10. Reingold EM, Stampe DM (1999) Saccadic inhibition in complex visual tasks. In: Current oculomotor research. Springer, pp 249–255

11. Goetz CG, Stebbins GT, Tilley BC (2012) Calibration of unified Parkinson’s disease rating scale scores to Movement Disorder Society-unified Parkinson’s disease rating scale scores. Mov Disord 27:1239–1242. https://doi.org/10.1002/mds.25122
