# Supplementary figures and images for: Eye tracking identifies biomarkers in α-synucleinopathies versus progressive supranuclear palsy
Source: J Neurol. 2022 Apr 30;269(9):4920–38. doi: 10.1007/s00415-022-11136-5 (PMC9363304; doi:10.1007/s00415-022-11136-5)

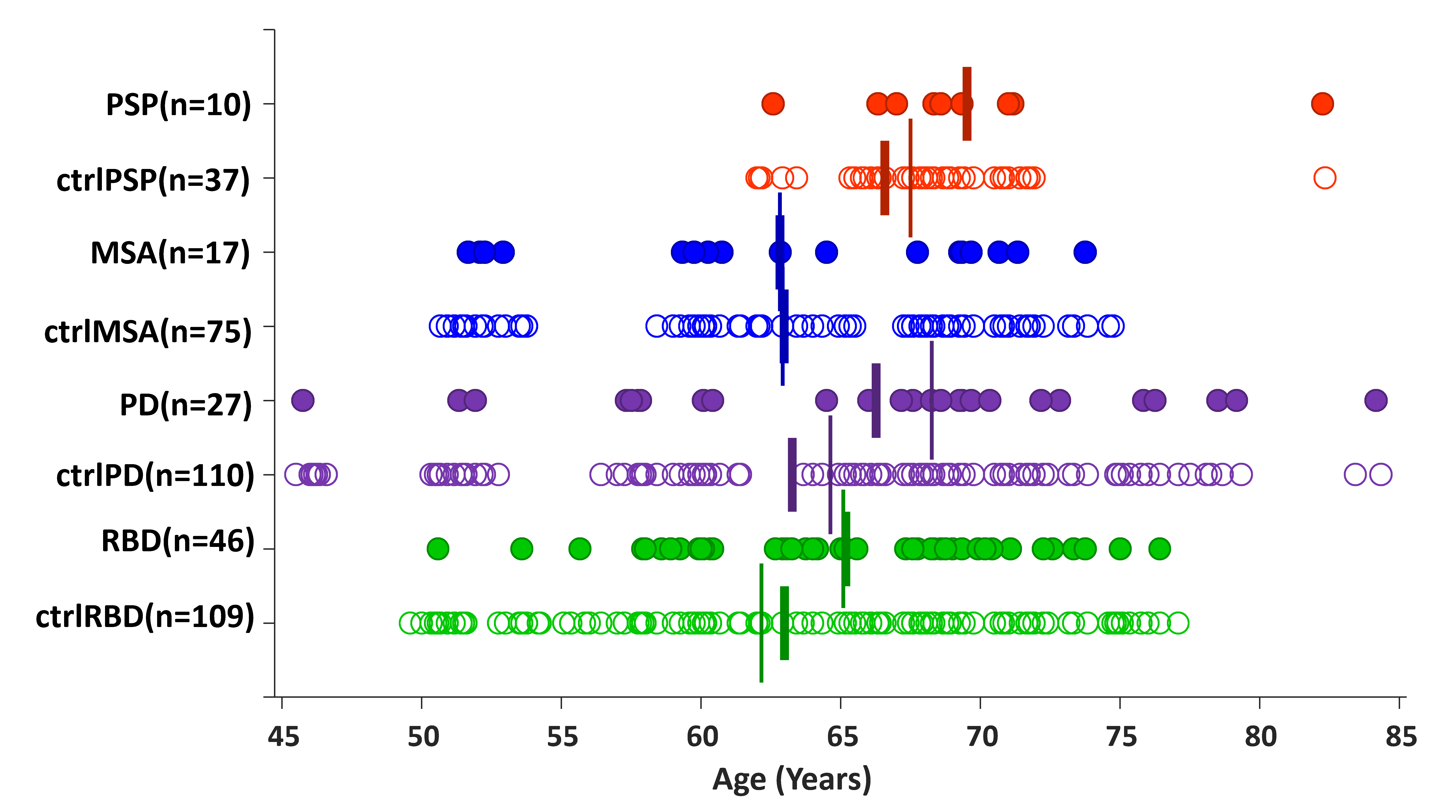

Supplement: Supplementary file 2 — Supplementary file2 (PNG 186 KB) [file 415_2022_11136_MOESM2_ESM.png]

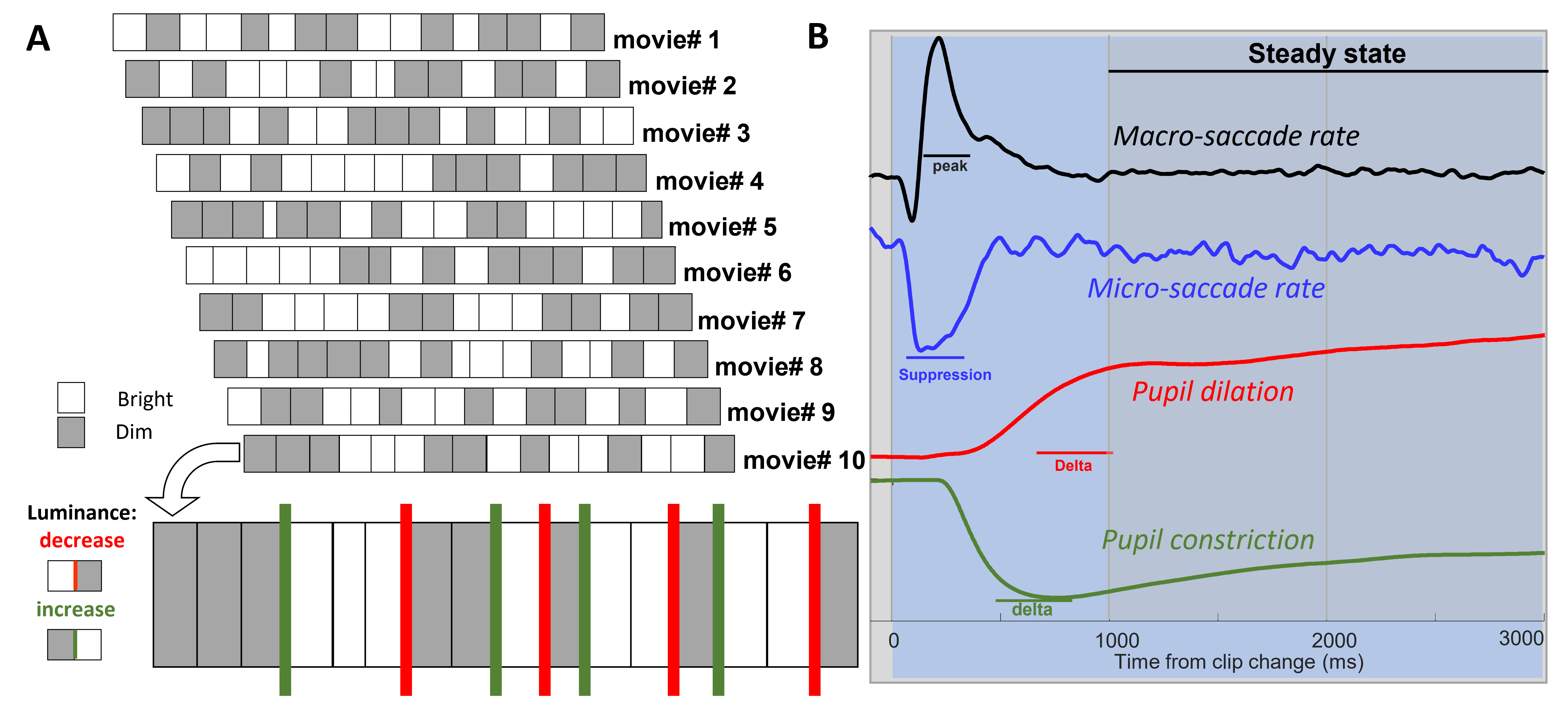

Supplement: Supplementary file 3 — Supplementary file3 (PNG 614 KB) [file 415_2022_11136_MOESM3_ESM.png]

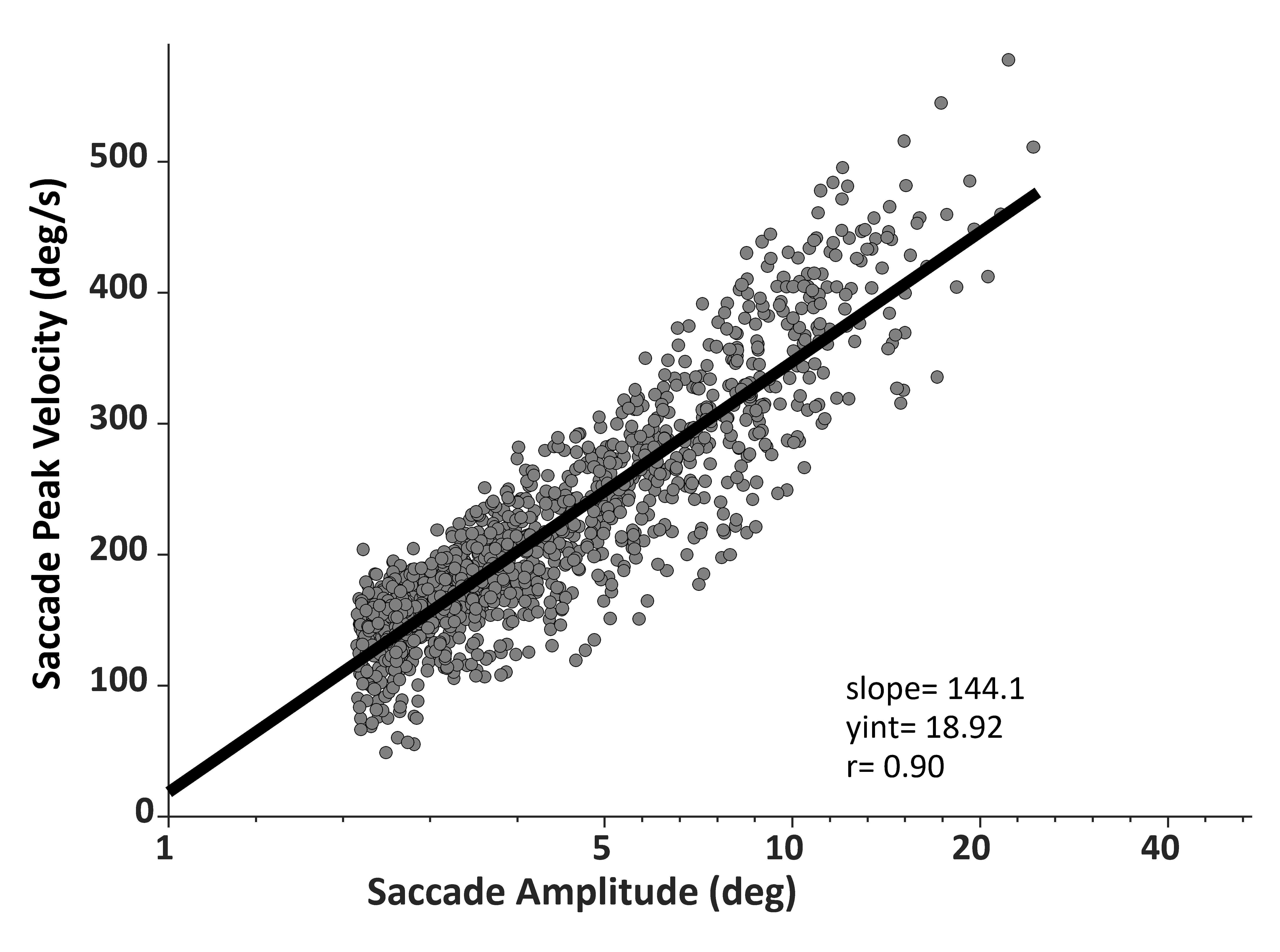

Supplement: Supplementary file 4 — Supplementary file4 (PNG 168 KB) [file 415_2022_11136_MOESM4_ESM.png]

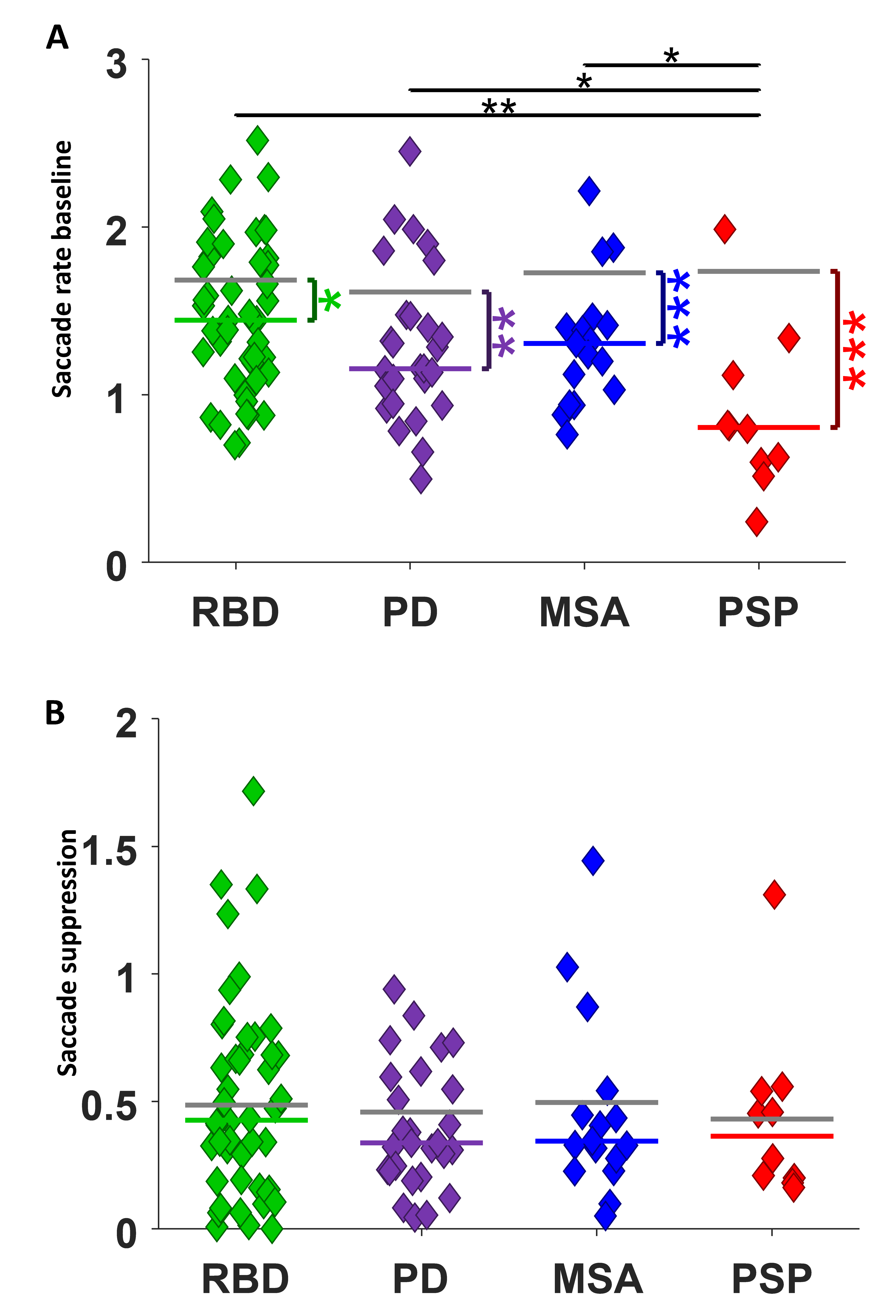

Supplement: Supplementary file 5 — Supplementary file5 (PNG 1010 KB) [file 415_2022_11136_MOESM5_ESM.png]

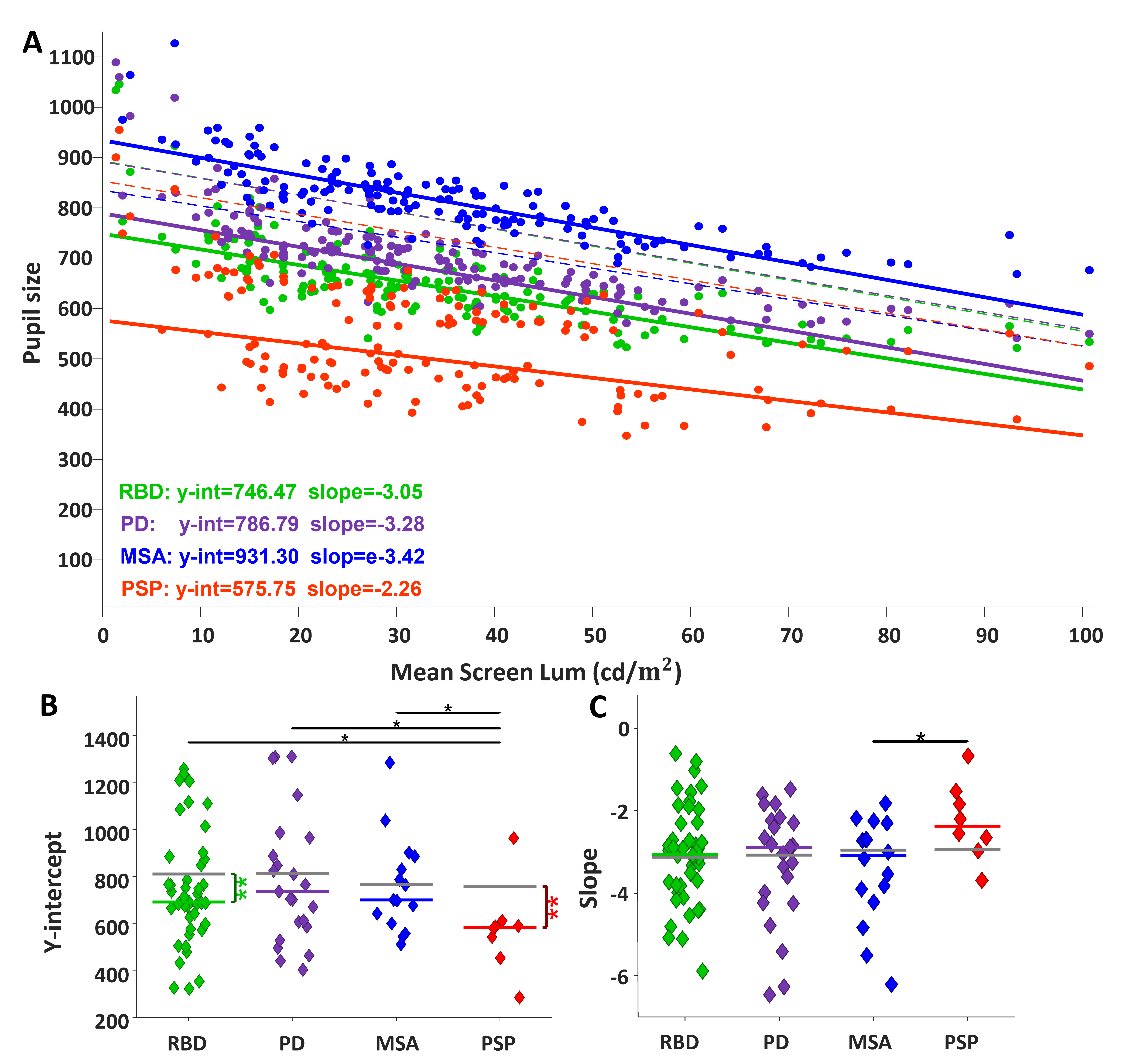

Supplement: Supplementary file 6 — Supplementary file6 (PNG 1556 KB) [file 415_2022_11136_MOESM6_ESM.png]

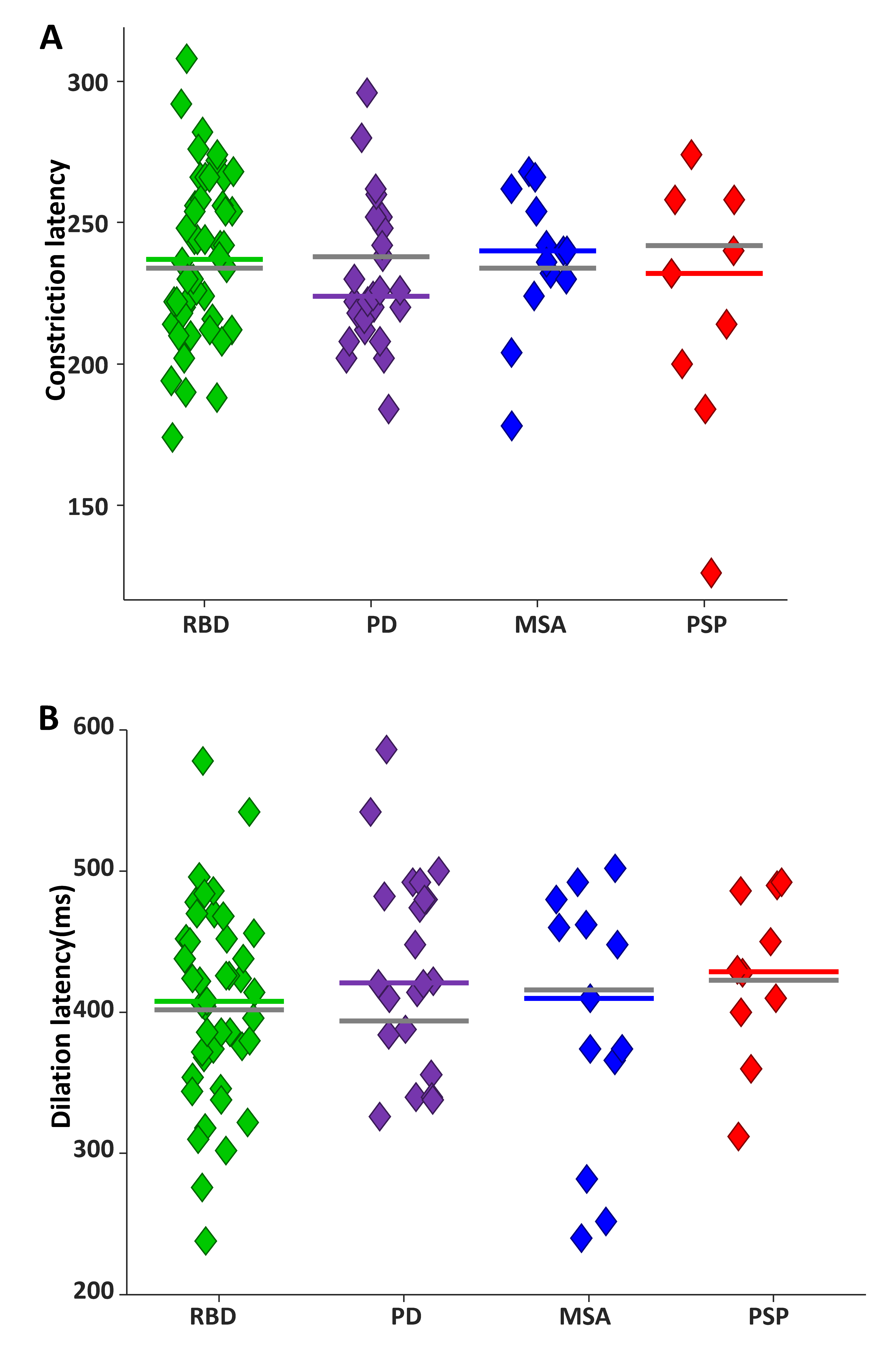

Supplement: Supplementary file 7 — Supplementary file7 (PNG 212 KB) [file 415_2022_11136_MOESM7_ESM.png]

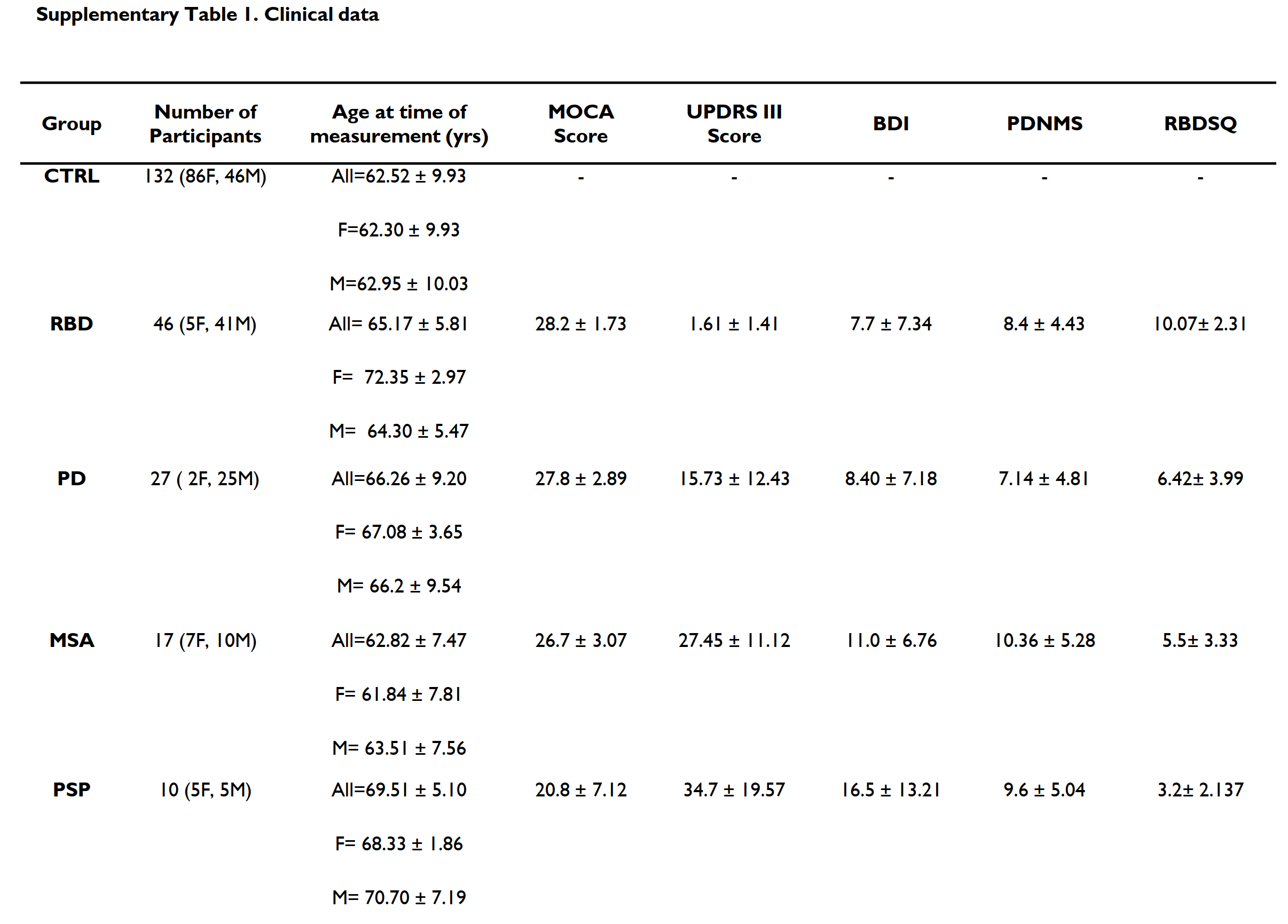

Supplement: Supplementary file 10 — Supplementary file10 (PNG 193 KB) [file 415_2022_11136_MOESM10_ESM.png]
